# Synthetic Linear Lipopeptides and Lipopeptoids Induce Apoptosis and Oxidative Stress: In Vitro Cytotoxicity and SAR Evaluation Against Cancer Cell Lines

**DOI:** 10.3390/ph18121840

**Published:** 2025-12-02

**Authors:** Ali Hmedat, Sebastian Stark, Tuvshinjargal Budragchaa, Nebojša Đ. Pantelić, Ludger A. Wessjohann, Goran N. Kaluđerović

**Affiliations:** 1 Department of Engineering and Natural Sciences, University of Applied Sciences Merseburg, Eberhard-Leibnitz-Strasse 2, 06217 Merseburg, Germany; ali.hmedat@yu.edu.jo; 2 Department of Pharmaceutics and Pharmaceutical Technology, Faculty of Pharmacy, Yarmouk University, Irbid 21163, Jordan; 3 Department of Bioorganic Chemistry, Leibniz Institute of Plant Biochemistry, Weinberg 3, 06120 Halle (Saale), Germany; starksebastian82@gmail.com (S.S.); tuvshinjargal.budragchaa@ipb-halle.de (T.B.); 4 Department of Chemistry and Biochemistry, Faculty of Agriculture, University of Belgrade, Nemanjina 6, 11080 Belgrade, Serbia; pantelic@agrif.bg.ac.rs

**Keywords:** lipopeptides, cytotoxicity, apoptosis, autophagy, caspase, structure–activity relationship (SAR)

## Abstract

**Background**: Cancer remains a major global health challenge, with current therapies often limited by high toxicity and poor selectivity. Lipopeptides, due to their amphiphilic architecture and synthetic accessibility, have emerged as promising anticancer agents. In this study, the in vitro cytotoxic potential and structure–activity relationships (SARs) of a library of 60 synthetic linear lipopeptides (LLPs), including lipopeptide–peptoid chimeras generated via the Ugi four-component reaction, were evaluated against four cancer cell lines (B16F10, HeLa, HT-29, and PC3). **Methods**: Cytotoxicity was assessed using MTT and crystal violet (CV) assays, and the natural cyclic lipopeptide surfactin was included as a reference. SAR analysis explored the effects of C-terminal functional groups, lipophilic tail length, peptide core size, and side chain modifications. Mechanistic studies involved cell cycle analysis, apoptosis markers (Annexin V/PI staining, caspase-3 activation), and oxidative stress assessment (ROS/RNS and NO production). **Results:** Several synthetic LLPs showed potent and selective anticancer activity, with IC_50_ values approximately 3–15 times lower than that of surfactin and with minimal toxicity toward non-cancerous NIH3T3 fibroblasts. Key structural determinants for activity included the presence of a C-terminal ester group, a lipophilic tail of 14–19 carbon atoms, and a tetrapeptide core. LLPs containing phenyl or azide side chains further enhanced cytotoxicity in a cell line-dependent manner. Mechanistic investigations confirmed that active LLPs induce caspase-dependent apoptosis, cell cycle arrest, and oxidative stress. These findings highlight that the synthetic LLPs demonstrate high in vitro anticancer efficacy with favorable selectivity. **Conclusions:** Synthetic LLPs exhibit potent and selective anticancer activity in vitro. SAR insights and mechanistic findings support their development as next-generation lipopeptide-based therapeutics.

## 1. Introduction

Cancer remains one of the most pressing global health challenges and has surpassed cardiovascular disease as the leading cause of premature mortality in many regions [1]. According to the latest estimates from the International Agency for Research on Cancer (IARC), nearly 20 million new cancer cases and 9.7 million cancer-related deaths were recorded globally in 2022 [2]. Although conventional treatments such as chemotherapy remain the cornerstone of cancer management, their clinical utility is often limited by dose-dependent toxicity, acquired drug resistance, and lack of tumor specificity [3,4,5]. These limitations highlight the urgent need for novel therapeutic strategies that offer improved efficacy with reduced systemic toxicity. In response, considerable research efforts have been devoted to identifying new molecular targets and developing structurally diverse compounds with enhanced anticancer properties [6,7,8]. Among these, lipopeptides (LPs) have emerged as particularly attractive candidates for investigation due to their potent cytotoxic potential and membrane-active behavior.

Lipopeptides are a class of low-molecular-weight amphiphilic molecules composed of a lipid moiety covalently linked to a short peptide chain, typically ranging from 4 to 12 amino acids. The lipid segment generally consists of a fatty acyl chain containing 12–18 carbon atoms, often attached to the N-terminus of a cyclic or linear peptide backbone [9]. These structural characteristics confer lipopeptides with amphipathic properties, enabling strong interactions with biological membranes and support a broad spectrum of bioactivities, including antimicrobial, immunomodulatory, and anticancer effects [10,11,12].

Naturally occurring LPs are biosynthesized by a wide range of microorganisms, including bacteria, fungi, and yeasts, and are often referred to as microbial surfactants [13]. These compounds serve as chemical defense agents, playing a critical role in protecting their producing organisms from predation by exerting toxic effects on surrounding cells [14,15]. Their biosynthesis, surface activity, low critical micelle concentration (CMC), and environmental biodegradability make them attractive candidates for diverse applications in food preservation, cosmetics, agriculture, and bioremediation [16,17]. Additionally, several LPs, including polymyxin B and daptomycin, have received FDA approval for clinical use due to their potent antimicrobial activity [18]. Beyond their well-established antimicrobial effects, LPs have also demonstrated significant anticancer, anti-inflammatory, and immunomodulatory activities [19,20]. These effects are primarily attributed to their ability to disrupt cell membranes, interfere with membrane potential, form transmembrane pores, and modulate intracellular signaling pathways involved in cell survival and apoptosis [21,22].

While many natural cyclic lipopeptides—such as surfactin, iturin, and fengycin— and their analogs have demonstrated moderate anticancer activity, their clinical translation has been limited due to issues such as nonspecific toxicity, hemolysis, and poor tumor selectivity [23,24,25,26]. Notably, several naturally derived linear lipopeptides (LLPs), including fellutamides, mitsoamide, and somocystinamide A, have shown pronounced cytotoxic effects through mechanisms involving apoptosis induction, cell cycle arrest, and mitochondrial dysfunction [27,28,29]. Although natural products often exhibit promising biological activities, they also possess inherent limitations, including suboptimal pharmacokinetics and toxicity [6]. This has driven efforts to introduce structural modifications aimed at improving therapeutic efficacy and safety profiles. In this context, LLPs are particularly attractive due to their synthetic flexibility and structural diversity, which make them excellent scaffolds for the rational design of selective and potent anticancer agents [30]. Despite their potential, comprehensive structure–activity relationship (SAR) studies on LLPs remain limited in the literature. To address this gap, the Ugi four-component reaction (Ugi-4CR) has emerged as a powerful synthetic strategy for the rapid generation of chemically diverse LLP libraries [31,32]. This multicomponent reaction enables the modular incorporation of varied building blocks, facilitating the systematic investigation of structural features and their influence on biological activity. At the Leibniz Institute of Plant Biochemistry (IPB), Halle, Germany, the Ugi-4CR has been successfully employed to synthesize a large library of synthetic LLPs for SAR-driven anticancer screening.

This work represents one of the first systematic evaluations of synthetic linear lipopeptides and lipopeptoids, linking specific structural features to their cytotoxic and mechanistic profiles across multiple cancer cell lines. In this study, we evaluate the in vitro cytotoxicity of 60 synthetic LLPs (peptidomimetics) rapidly synthesized via Ugi-4CR against four human cancer cell lines (B16F10, HeLa, HT-29, and PC3), with surfactin serving as a natural reference. We aimed to identify structural motifs associated with enhanced anticancer activity, using SAR analysis to determine the influence of lipophilic tail length, terminal functionalities, peptide core size, and side chain composition. Mechanistic studies were conducted to elucidate the roles of apoptosis, oxidative stress, and antiproliferative effects in LLP-induced cytotoxicity. These findings lay the foundation for the rational design of next-generation LLP-based therapeutics with improved efficacy and safety profiles.

## 2. Results

### 2.1. Cytotoxic Activity of LLPs

A total of 60 synthetic linear lipopeptides (LLPs) were retrieved from the IPB-NWC compound library for evaluation of their cytotoxic potential. Their chemical structures, used for candidate selection, are shown in Figures S1 and S2, and summarized in Table S1. Candidate selection from the database was based on structural features associated with cytotoxic activity observed during initial screening. These features included the length of the lipophilic tail, the nature of the C-terminus (ester or carboxylic acid functional group), the number of amino acids in the peptidic core, the number and length of branches, and the nature of the branched functional groups (e.g., Boc or others). Collectively, these parameters were identified as critical contributors of the cytotoxic activity of active LLPs. This systematic approach ensured the inclusion of both highly active and moderately active LLPs for subsequent SAR analysis.

**Figure 1:**
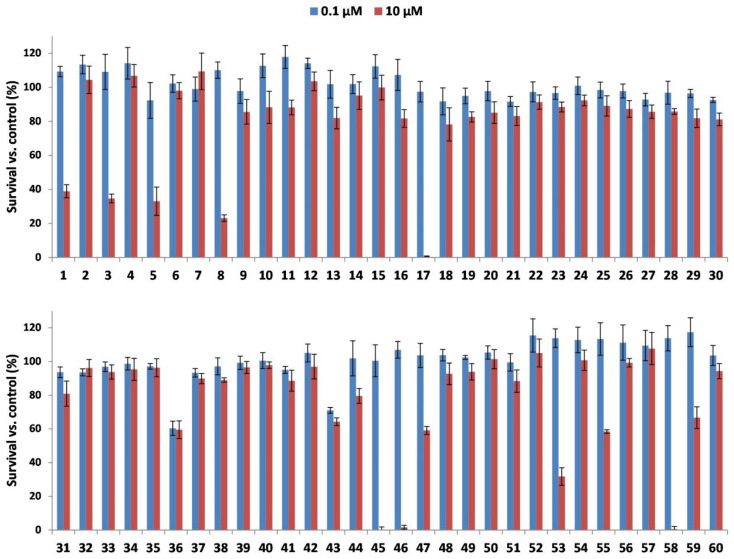
Cytotoxic effects of the investigated LLPs on the B16F10 cell line, as assessed by the CV assay. Cells were treated with DMSO (vehicle control) or with 0.1 µM and 10 µM concentrations of each LLP for 72 h. Cell viability was normalized to the DMSO-treated control. Data represent the mean ± SD of three independent biological replicates.

All selected LLPs were screened for cytotoxic effects against four cancer cell lines: B16F10 (mouse skin melanoma), HeLa (human cervical adenocarcinoma), HT-29 (colon adenocarcinoma), and PC3 (prostate cancer) cell lines using MTT and CV assays. These cell lines were chosen to represent distinct cancer types, allowing a broader evaluation of LLP cytotoxicity across different tumor models. The fast screening was performed at two concentrations of LLPs (0.1 and 10 μM) after 72 h of treatment. The cytotoxic effects of LLPs on B16F10 cells, assessed by the CV assay, are presented in Figure 1. For corresponding MTT assay results and data for the other cell lines, refer to Supplementary Figures S3–S9. Notably, a considerable number of LLPs demonstrated significant cytotoxic activity against the investigated cancer cell lines at 10 μM concentration.

Compounds exhibiting cytotoxic activity at 10 μM or lower were selected for further investigation, while less active compounds were also evaluated to enable a comprehensive structure–activity relationship (SAR) analysis. Selected active compounds, along with surfactin as a reference lipopeptide, were further tested across a concentration range of 0.625–80 μM to determine their half-maximal inhibitory concentration (IC_50_), defined as the concentration required to inhibit 50% of cell viability. IC_50_ values of the LLPs against all tested cancer cell lines are presented in Table 1 and represent the mean of three independent experiments using CV assay (corresponding MTT data are provided in Supplementary Materials, Table S2). Dose–response curves for representative LLPs and surfactin are shown in Figure S10 to illustrate their concentration-dependent cytotoxic effects. Notably, results from the MTT and CV assays were consistent, indicating that LLPs and surfactin do not compromise cellular respiration [33,34]. The MTT assay measures cell viability based on mitochondrial metabolic activity, while the CV assay quantifies the total number of adherent cells by staining their DNA and proteins. Together, they provide complementary information on both metabolic status and overall cell mass. These findings clearly demonstrate that synthetic LLPs outperform the natural cyclic lipopeptide surfactin in terms of cytotoxic potency. Overall, all investigated LLPs demonstrated markedly higher anticancer activity compared to surfactin, with IC_50_ values approximately 3–15 times lower across the investigated cancer cell lines. An exception was LLP **48**, which showed no activity against B16F10 and PC3 cell lines.

### 2.2. Evaluation of Cytotoxic Selectivity Against Normal Fibroblasts

**Figure 2:**
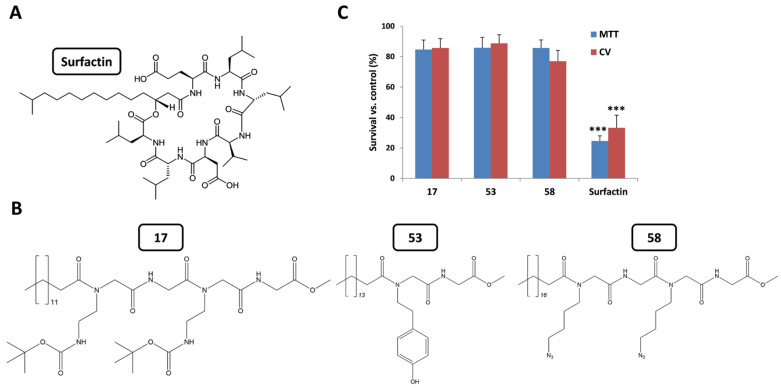
(**A**) Surfactin structure. (**B**) Chemical structures of LLPs **17**, **53** and **58**. (**C**) Survival of NIH3T3 fibroblasts treated with LLPs or surfactin at IC_50_ concentrations for 72 h, determined by MTT and CV assays. Cell viability values were normalized to DMSO-treated control cells and are presented as mean ± SD from three independent experiments. Statistical significance versus control was determined using a two-tailed *t*-test; *** *p* < 0.001.

Surfactin (Figure 2A) was included in subsequent evaluations to compare the selectivity profiles of natural and synthetic lipopeptides (LPs) toward normal cells. Three active LLPs (compounds **17**, **53** and **58**) were selected for this evaluation, representing LLPs containing Boc, phenol, and azide functional groups in their side chains, respectively (Figure 2B). These LLPs, along with surfactin, were tested on mouse embryonic NIH3T3 fibroblasts at their predetermined IC_50_ concentrations to assess their effects on non-cancerous cells. The NIH3T3 fibroblast cell line was included as a non-cancerous control because it is a well-established, robust model for assessing compound selectivity and cytotoxicity toward normal cells [37,38]. MTT and CV assay results demonstrated that surfactin exhibits a markedly higher toxicity toward NIH3T3 cells compared to the active LLPs (Figure 2C). Treatment with surfactin at its IC_50_ concentration reduced NIH3T3 cell viability to approximately 25%, indicating substantial toxicity to normal cells. In contrast, treatment with active LLPs resulted in high cell viability (80–90%), indicating that these synthetic compounds selectively targeted cancer cells with negligible or no cytotoxic effects on normal fibroblasts. These observations underscore the favorable selectivity profiles of the synthetic LLPs compared to the natural lipopeptide surfactin.

### 2.3. Structure–Activity Relationship (SAR) Analysis

Several LLPs exhibited unexpectedly high anticancer activity across the tested cell lines, highlighting the importance of conducting a systematic SAR analysis. Following the evaluation of cytotoxic selectivity, a detailed SAR analysis was performed to identify the key structural features governing the cytotoxic activity of the investigated LLPs. In the present study, cell survival results from both MTT and CV assays were employed to delineate the structural determinants responsible for the biological activity of the LLPs.

#### 2.3.1. Effect of the C-Terminus Functional Group on Cytotoxic Activity

A critical role for the *C*-terminus functional group in modulating anticancer activity was observed. Active compounds predominantly contained an ester functional group at the *C*-terminus, whereas LLPs featuring a carboxylic acid group were largely inactive. Out of the seven compounds containing a carboxylic acid terminus (Figure S2), none exhibited significant cytotoxicity against the investigated cancer cell lines (Figure 1 and Figures S3–S9). The ester group, being neutral and more lipophilic than the negatively charged carboxyl group, likely enhances cell membrane penetration and thus contributes to the cytotoxic effects [39,40]. These findings underscore the significance of C-terminal esterification in enhancing the anticancer activity of LLPs.

#### 2.3.2. Effect of Lipophilic Tail Length on the Cytotoxic Activity

A nonlinear relationship was noted between the number of carbon atoms in the lipophilic tail and the cytotoxicity of LLPs, as observed through the investigation of a homologous series of synthetic compounds (Figure S11). Compounds **35** through **44** share an identical core structure but differ in fatty acid chain length (9–18 carbon atoms), demonstrated a clear trend: compounds with the longest fatty acid chains (**43** and **44**) exhibited the highest cytotoxic activity across all investigated cell lines.

**Figure 3:**
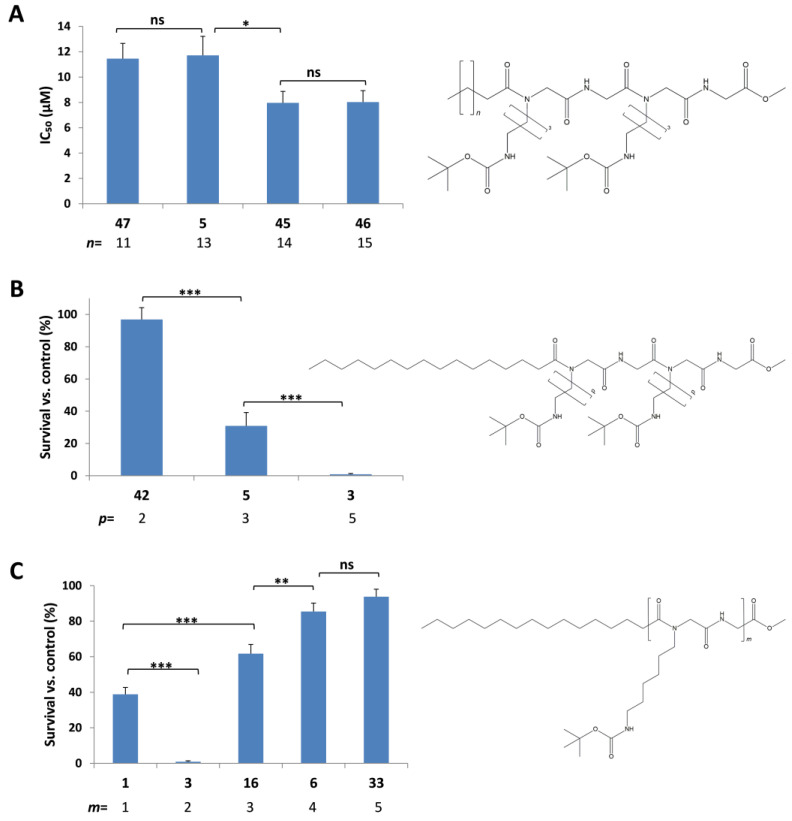
(**A**) Correlation between the IC_50_ values and the fatty acid chain length of LLPs **47**, **5**, **45**, and **46**, as determined by the CV assay. B16F10 cells were treated with serial dilutions of the investigated LLPs for 72 h. *n* = number of CH_2_ groups in the fatty acid chain. IC_50_ values are presented as mean ± SD from three independent experiments. (**B**) Association between B16F10 cell survival and the side chain length of LLPs **42**, **5**, and **3**, assessed by CV assay. B16F10 cells were treated with 10 μM of each LLP for 72 h. *p* = number of CH_2_ groups in the alkyl side chain. (**C**) Correlation between cell survival of B16F10 cells after 72 h treatment with 10 μM LLPs (CV assay) and the number of dipeptoid repetitions in the LLPs **1**, **3**, **16**, **6**, and **33**. *m* = number of dipeptoids (i.e., 2 *m* = number of amino acids in the chain). (**B**,**C**) Values are normalized to DMSO-treated cells and are presented as mean ± SD from three independent experiments. Statistical significance was assessed using a two-tailed *t*-test. Asterisks indicate significance levels: ns = not significant; * *p* < 0.05; ** *p* < 0.01; *** *p* < 0.001.

To further confirm the relationship between fatty acid chain length and cytotoxicity, additional structurally related LLPs of the (peptoid–peptide) oligomer type with varying fatty acid chain lengths were analyzed in detail. Compounds **47**, **5**, **45**, and **46**, which share an identical central scaffold but differ in their lipophilic tail lengths (14, 16, 17, and 18 carbon atoms, respectively; Figure 3A), exhibited significant cytotoxicity against the investigated cancer cell lines (Figure 1 and Figures S3–S9). In contrast, structurally similar LLPs **28**, **29**, and **30**, bearing shorter lipophilic chains of 10, 11, and 12 carbon atoms, respectively (Figure S1), showed no detectable cytotoxic activity across the tested cell lines (Figure 1 and Figures S3–S9). A marked enhancement in anticancer activity was observed when increasing the fatty acid chain length from inactive C10–C12 LLPs to active C14–C18 LLPs. To establish a more predictive relationship between lipophilic tail length and cytotoxicity, IC_50_ values of compounds **47**, **5**, **45**, and **46** were determined (Table 1). Notably, compounds **45** and **46**, containing 17 and 18 carbon atoms in their fatty acid chains, displayed the lowest IC_50_ values, approximately 8 µM, across the investigated cancer cell lines (Table 1 and Figure 3A).

Consistent with the observed trend, the cytotoxic profile continued to improve with increasing lipophilic chain length up to approximately 19 carbon atoms; however, further extension beyond this point did not substantially enhance cytotoxicity. This saturation effect was evident from IC_50_ comparisons among LLPs **55**, **58**, and **59**, which share an identical core structure but differ in their fatty acid tail lengths (16, 19, and 20 carbon atoms, respectively; Figure S2). The IC_50_ value decreased when extending the tail from 16 carbon atoms in compound 55 to 19 carbon atoms in compound 58, reflecting improved cytotoxic activity. However, a further increase in tail length to 20 carbon atoms in compound 59 did not lead to any additional improvement in cytotoxic potency; instead, an increase in IC_50_ values was observed against the tested cancer cell lines (Table 1). It is possible that the decreased activity shown in LLPs with lipophilic tails longer than 19 carbon atoms is due to their higher hydrophobicity, which causes them to self-aggregate in aqueous environments. This, in turn, limits their solubility, membrane partitioning, and the efficiency of cellular uptake.

#### 2.3.3. Effect of Alkyl Side Chain Length on the Cytotoxic Activity

The absence of an absolute correlation between lipophilic tail length and cytotoxicity suggested that additional structural parameters could also influence the cytotoxic response of LLPs. To explore this possibility, the effect of alkyl side chain length in substituted amino acids was investigated by selecting a set of LLPs sharing an identical lipophilic tail and peptidic core. Compounds **42**, **5**, and **3** each contain a tetrapeptide scaffold with two *N*-substituted amino acids (peptoid moieties), differing only in the length of the alkyl side chains: propylene, butylene, and hexylene, respectively. The cytotoxic effects of these compounds are presented in Figure 3B. It is clearly observed that increasing the number of carbon atoms in the alkyl side chain led to significant improvements in cytotoxic activity. This enhanced potency may be attributed to the overall rise in compound hydrophobicity associated with the elongation of the alkyl side chains.

#### 2.3.4. Effect of Side Chain Functional Groups on the Cytotoxic Activity

In addition to alkyl side chain length, the nature of the functional groups attached to the side chains was also found to significantly influence the cytotoxic activity of LLPs. The most active LLPs were those still containing a Boc (*tert*-butoxycarbonyl) protecting group (Figure S1 and Table 1). Several studies have demonstrated that the incorporation of protected amino acid groups can enhance both the antitumor activity and selectivity [41,42]. Selective modification of the Boc side chain during synthesis, by replacement with other functional groups, generally resulted in a complete or partial loss of cytotoxic potency, except for compounds incorporating phenyl or azide moieties (Table S1 and Figure S2).

For instance, compound **1**, which carries a Boc group, exhibited an IC_50_ value of approximately 12 μM against the B16F10 cell line (Table 1). Substitution of the Boc group with an indole group (LLP **50**, Table S1) or an azide (LLP **52**, Figure S2) led to a significant reduction in anticancer activity against B16F10 cells (Figure 1 and Figure S3). Conversely, the introduction of a 4-hydroxyphenyl group in compound **53** (Table S1) enhanced the cytotoxic response, lowering the IC_50_ value to 7.7 μM against B16F10 cells (Table 1). A similar trend was observed in the HT-29 and PC3 cell lines, where compound **53** exhibited higher cytotoxic activity compared to compound **1**. These findings underscore that certain aromatic/phenolic or electron-rich functionalities can maintain or even enhance biological activity within an appropriate structural framework.

#### 2.3.5. Effect of Peptidic Core Length on the Cytotoxic Activity

In this study, the investigated LLPs contained between 2 and 10 amino acids. The length (repetition number) of the peptoid–peptidic core was found to play a critical role for the cytotoxic potency. Figure 3C presents the survival rates of B16F10 cells treated with synthetic LLPs that share the same lipophilic tails, protective groups, and alkyl side chain lengths but differ in the number of amino acids in their core structures (2, 4, 6, 8, and 10 amino acids, corresponding to compounds **1**, **3**, **16**, **6**, and **33**, respectively). Based on these results, LLPs containing four amino acids, i.e., 2 (peptoid–peptide) repeats, exhibited the highest cytotoxic activity (Figure 3C). In contrast, compounds containing eight or ten amino acids showed no significant cytotoxic effects. If this is due to their larger size, which may hinder membrane penetration, is unclear, as for oligolysin/arginin penetration longer the optimum is with longer peptides [43]. Similar trends were also observed across other tested cancer cell lines (Figure S12). These findings suggest that an optimal balance among key structural features, including lipophilic tail length, alkyl side chain length, and functional group type, must be achieved to maximize anticancer activity. Statistical comparisons among LLP structural variants confirmed that differences in cytotoxic activity correlated significantly with variations in lipophilic tail length, alkyl side chain length and functionality, and peptide core size (Figure 3A–C, Figures S11 and S12). The optimal structural balance was most clearly observed in four amino acid LLPs, which demonstrated strong cytotoxicity across a wide range of fatty acid chain lengths (13–18 carbon atoms) and various side chain modifications.

### 2.4. Mechanism of Action

#### 2.4.1. Induction of sub-G1 Cell Cycle Arrest

**Figure 4:**
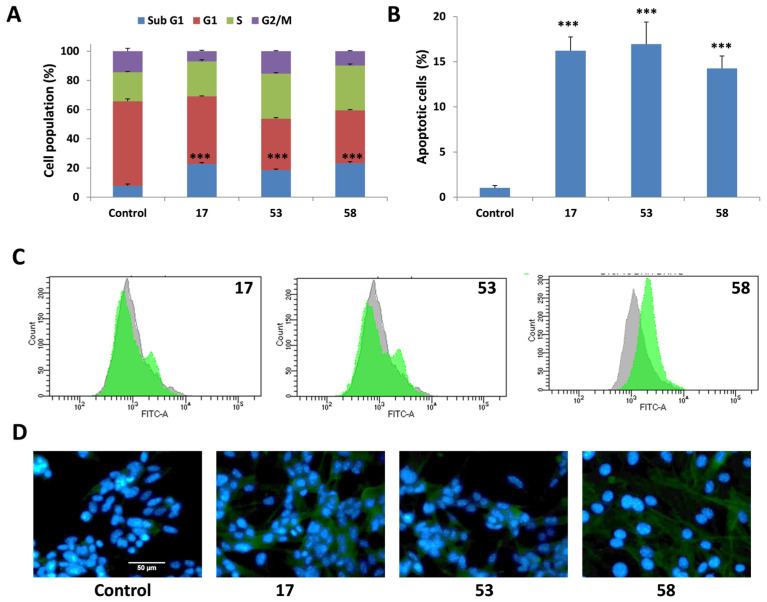
(**A**) Cell cycle distribution of B16F10 cells treated with LLPs **17**, **53** or **58** for 72 h at IC_50_ concentrations, assessed by DAPI staining. (**B**) Induction of apoptosis in B16F10 cells following treatment with selected active LLPs. Apoptosis was assessed using Annexin V/PI staining after 72 h of treatment with active LLPs at their respective IC_50_ concentrations. In (**A**,**B**), values are shown as mean ± SD from three independent experiments, and statistical significance versus control was determined using a two-tailed *t*-test (*p* < 0.001, ***). (**C**) Caspase activity in B16F10 cells following treatment with selected active LLPs (ApoStat, IC_50_, 72 h). Histograms display caspase-dependent fluorescence, with gray indicating untreated control cells and green representing LLP-treated cells. Data shown are representative of three independent experiments. (**D**) Fluorescence images (40×) showing caspase-3 activation in B16F10 cells after 72 h treatment with LLPs at IC_50_ concentration. Nuclei are stained with DAPI (blue), and caspase-3 activation is visualized using a primary rabbit anti-caspase-3 antibody and an Alexa Fluor 488-conjugated secondary antibody (green). Data are representative of three independent experiments.

To gain insights into the possible mechanisms underlying the cytotoxic effects of active LLPs, flow cytometry analysis was performed to examine cell cycle distribution following treatment. B16F10 cells were exposed to three selected active LLPs (compounds **17**, **53** and **58**, representing Boc-, phenol- and azide-containing LLPs, respectively) at their IC_50_ concentrations for 72 h, and DNA content was assessed using DAPI staining. As shown in Figure 4A, treatment with LLPs led to a notable accumulation of cells in the sub-G1 phase (approximately 20%). This effect was more pronounced when cells were treated with double IC_50_ concentrations for 72 h (50–60%), suggesting a dose-dependent induction of apoptotic pathways (Figure S13A). The accumulation of cells in the sub-G1 phase is a hallmark of apoptosis, reflecting the presence of fragmented DNA due to the activation of cell death signaling cascades [44,45]. These initial findings prompted further investigations into the apoptotic mechanisms induced by LLPs.

#### 2.4.2. Induction of Apoptosis Confirmed by Annexin v/PI Staining

To further confirm whether the cytotoxic effects of LLPs involved apoptotic cell death, Annexin V-FITC and PI double staining followed by flow cytometry analysis was performed. B16F10 cells were treated with the selected active LLPs (**17**, **53**, and **58**) at their IC_50_ concentrations for 72 h. As shown in Figure 4B, treatment with LLPs resulted in a significant increase in the proportion of apoptotic cells compared to untreated controls, reaching approximately 15%. These results confirm that apoptosis is a possible mode of cell death induced by the active LLPs in B16F10 cells and support the observations from the sub-G1 cell cycle analysis.

#### 2.4.3. Activation of Caspases During LLP-Induced Apoptosis

Apoptosis is frequently mediated by proteolytic enzymes known as caspases, which trigger cell death by cleaving specific substrates within the cytoplasm and nucleus [46]. Therefore, in the current study, flow cytometry analysis using the ApoStat assay was employed to identify caspase activity in B16F10 cells treated with active LLPs. In this assay, apoptotic cells are irreversibly labeled with a cell-permeable, FITC-conjugated pan-caspase inhibitor (ApoStat), and increased fluorescence indicates the activation of caspases [47].

Cells treated with compounds **17** and **53** at their IC_50_ concentrations for 72 h showed a gradual enhancement in the fluorescence signal, evidenced by the formation of a distinct secondary peak, confirming the activation of caspases during the apoptotic process (Figure 4C). In the case of compound **58**, clearer caspase activation was detected, characterized by a shift in the fluorescence peak compared to the control.

Additionally, immunocytochemistry analysis was performed to further validate caspase-3 activation. B16F10 cells treated with active LLPs showed enhanced caspase-3 activity as visualized by fluorescence microscopy using the EVOS FL Auto Imaging System (Figure 4D), reinforcing the involvement of the caspase cascade in LLP-induced apoptosis.

#### 2.4.4. Lack of Autophagy Induction

Autophagy is a cellular process that can contribute to cell death through the autophagosomal–lysosomal degradation of intracellular components [48]. To evaluate the potential involvement of autophagy in LLP-induced cytotoxicity, acridine orange (AO) staining was performed to assess the formation of acidic vesicular organelles in B16F10 cells treated with selected active LLPs. As shown in Figure S13B, no significant increase in acidic vesicle formation was detected following treatment with any of the examined LLPs. These findings suggest that autophagy does not substantially contribute to the cytotoxic mechanisms of the investigated compounds.

#### 2.4.5. Effect of LLPs on Cell Proliferation

**Figure 5:**
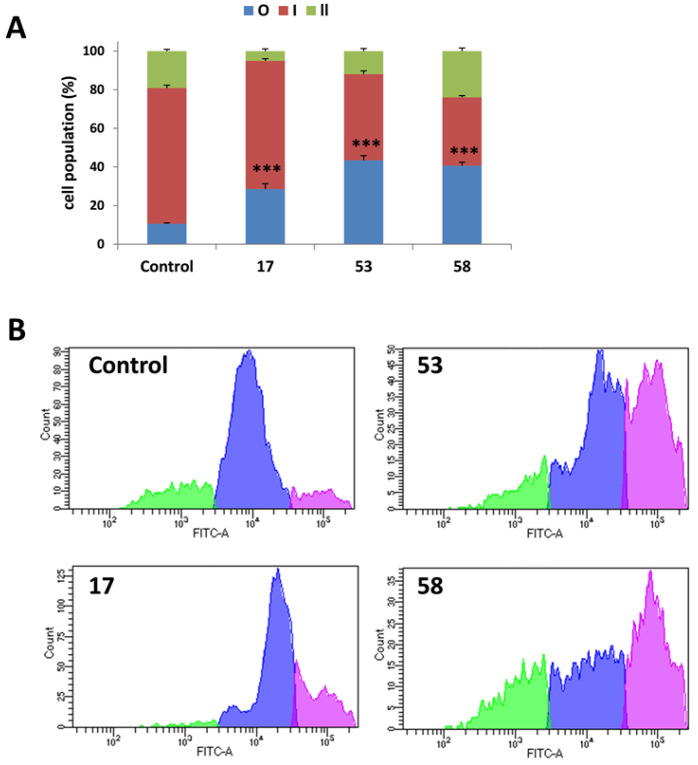
(**A**) Proliferation profiles of B16F10 cells stained with CFSE and treated for 72 h at the IC_50_ concentrations of **17, 53** and **58**. O indicates undivided cells; I and II represent the first and second cell divisions, respectively. Values are presented as mean ± SD from three independent experiments, and statistical significance versus control was determined using a two-tailed *t*-test (*** *p* < 0.001). (**B**) Representative FACS images showing the proliferative profile of control and LLP-treated B16F10 cells (IC_50_, 72 h) stained with CFSE. Pink, blue, and green peaks correspond to the first, second, and third cell generations, respectively. Displayed here are representative of three independent experiments.

The impact of active LLPs on the proliferative capacity of B16F10 cells was evaluated using a CFSE (carboxyfluorescein succinimidyl ester) cell proliferation assay. B16F10 cells were stained with CFSE and subsequently treated with either compound **17**, **53** or **58** at their IC_50_ concentrations for 72 h. Flow cytometry analysis revealed a significant alteration in the cell division profile compared to untreated controls (Figure 5A). In control cells, three distinct generations could be recognized after four days of cultivation, with the majority of the cell population successfully progressing through one to two cell divisions during the course of the experiment (Figure 5B). In contrast, treatment with active LLPs notably reduced the proportion of cells undergoing successive divisions, resulting in a higher percentage of cells remaining in the first generation (undivided). This effect was more pronounced in B16F10 cells treated with LLPs containing phenol **53** and azide **58** functional groups. These findings indicate that active LLPs impair the proliferative ability of B16F10 cells, contributing to their overall antiproliferative and cytotoxic effects.

#### 2.4.6. Generation of Reactive Oxygen and Nitrogen Species (ROS/RNS) and Nitric Oxide (NO)

ROS/RNS and NO can contribute to cell death by reacting with essential cellular structures and molecules, thereby altering their biological function or forming toxic products [49,50,51]. To evaluate the potential involvement of ROS, RNS, and NO in LLP-induced cytotoxicity, flow cytometry analysis was performed. For this purpose, B16F10 cells were treated with selected active LLPs (compounds **17**, **53**, and **58**) at their IC_50_ concentrations for 72 h and stained with DHR (for ROS/RNS detection) and DAF-FM (for NO detection).

**Figure 6:**
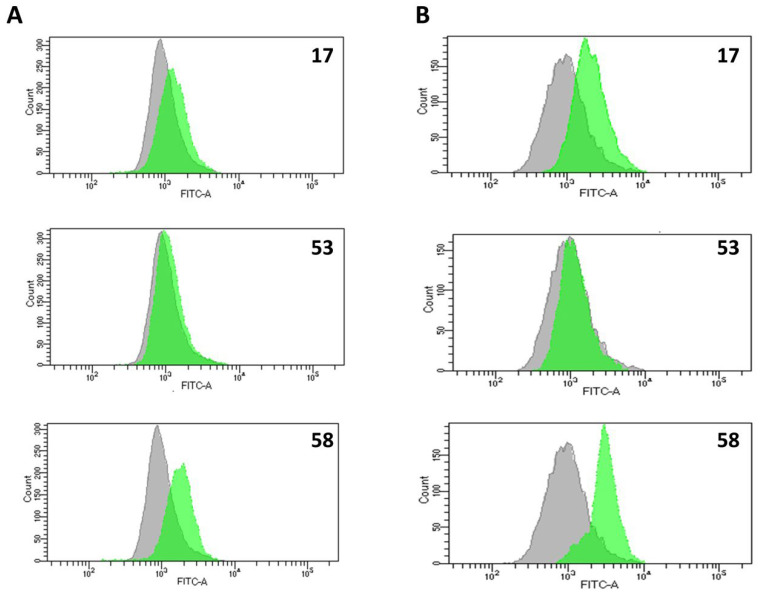
Flow cytometry analysis of (**A**) ROS/RNS production using DHR staining and (**B**) NO generation using DAF-FM staining in B16F10 cells treated with selected active LLPs. Cells were exposed to the IC_50_ concentration of each LLP for 72 h. In each histogram, gray represents the control signal, while green indicates the fluorescence signal from LLP-treated cells. (**A**,**B**) histograms are representative of three independent experiments.

As shown in Figure 6A, treatment with compound **58** (azide-containing LLP) led to a marked increase in ROS/RNS production compared to untreated controls. Compound **17** (Boc-containing LLP) also induced moderate ROS/RNS generation, whereas compound **53** (phenol-containing LLP) only slightly enhanced ROS/RNS levels relative to control cells. Similarly, increased NO production was observed in cells treated with compounds **17** and **58**, while compound **53** induced only a slight increase in NO levels (Figure 6B). These results suggest that the generation of ROS/RNS and NO may play a role in the cytotoxic mechanisms of specific LLPs, particularly those containing Boc and azide functional groups.

## 3. Discussion

In this study, we systematically evaluated the cytotoxic properties and underlying mechanisms of action of a panel of synthetic LLPs against multiple cancer cell lines, including B16F10, HeLa, HT-29, and PC3. Several LLPs demonstrated potent anticancer activity, with IC_50_ values approximately 3–15 times lower than the natural lipopeptide surfactin, underscoring their enhanced efficacy. Previous studies have shown that natural cyclic lipopeptides, including surfactin, iturin, fengycin, apratoxin, and pseudofactin, exhibit cytotoxic activity against various cancer cell lines [52,53,54,55,56]. Similarly, several naturally derived LLPs have demonstrated promising anticancer properties. Notably, fellutamides and somocystinamide A have shown variable cytotoxic effects across a range of cancer cell lines, including PC-3, Jurkat, HCT-15, A549, and SK-OV-3, through mechanisms involving cell cycle arrest, caspase-dependent apoptosis, and mitochondrial dysfunction [57,58,59]. However, the clinical utility of both cyclic and linear lipopeptides has often been constrained by inconsistent efficacy, nonspecific toxicity, and a limited understanding of their structure–activity relationships [35,55,56,60]. For instance, Bacillus-derived cyclic lipopeptides such as surfactin, fengycin, and iturin demonstrate varying degrees of hemolytic activity, which may limit their therapeutic index in clinical settings [61,62].

In our study, several synthetic LLPs demonstrated improved selectivity, exhibiting minimal cytotoxicity toward normal NIH3T3 fibroblasts at their IC_50_ concentrations, in contrast to surfactin, which significantly reduced normal cell viability. This selectivity may be linked to favorable structural features identified through our SAR analysis in our synthetic LLPs, such as specific functional groups, balanced hydrophobicity, and optimal peptide length, which likely contribute to improved discrimination between cancer and non-cancer cells. These insights highlight the potential of synthetic LLPs to serve as a new class of anticancer agents with enhanced efficacy and reduced safety profiles compared to their natural counterparts.

Structure–activity relationship analysis of the synthetic LLPs revealed that cytotoxic activity is strongly influenced by a combination of structural features, including the nature of the C-terminus, the length and composition of the lipophilic tail, N-side chain modifications, and the number of amino acids in the peptidic core. These observations are consistent with broader SAR principles established in lipopeptide research, where activity is modulated by amphipathicity, length and branching of the fatty acid chains and amino acid substitutions [63,64,65]. Previous studies have demonstrated similar effects in antimicrobial and antifungal lipopeptides, such as surfactins, iturins, and fengycins, where subtle modifications in acyl chain length or amino acid substitutions result in distinct biological profiles [66,67,68,69,70]. The concept of congeners, structural variants of a given lipopeptide differing in fatty acid length or acyl chains, has been widely reported among Bacillus and Pseudomonas species, with specific isoforms often exhibiting enhanced potency or altered selectivity [71,72,73]. While our study focused on synthetic analogs rather than microbial isolates, the structural diversity and functional trends observed among the LLPs strongly mirror those seen in natural lipopeptide congeners, highlighting the potential of rational design in optimizing anticancer bioactivity.

Notably, the presence of an ester group at the *C*-terminus was essential for cytotoxic activity, whereas LLPs terminating in a carboxylic acid group were inactive across all tested cancer cell lines. The increased negative polarity and reduced lipophilicity of carboxylic acid groups likely hinders cellular uptake by limiting passive membrane diffusion and intracellular accumulation [74]. The superior anticancer activity of ester-terminated LLPs may be attributed not only to their increased lipophilicity, which facilitates enhanced membrane penetration, but also to their potential to disrupt mitochondrial function. Previous studies have shown that esterified compounds can induce mitochondrial membrane depolarization, ATP depletion, and disruption of oxidative phosphorylation, ultimately leading to apoptotic cell death [75,76,77]. Such mitochondrial impairment may contribute to the selective cytotoxic effects observed with ester-containing LLPs, providing a reasonable mechanistic explanation for their enhanced potency compared to their free carboxylic acid counterparts.

A clear correlation between lipophilicity and cytotoxicity was observed, with LLPs containing 14–19 carbon atoms in their lipophilic chains exhibited the highest potency across tested cancer cell lines. This finding is consistent with previous studies showing that bioactive peptides, including antimicrobial and anticancer lipopeptides, must exceed a specific hydrophobicity threshold to effectively interact with and penetrate cellular membranes [78,79]. Our data suggest that a minimum lipophilic tail length of approximately 14 carbon atoms is required to elicit significant cytotoxic effects, with increasing chain length up to 19 carbons further enhancing activity. However, beyond this threshold, additional increases in lipophilicity did not translate into improved cytotoxicity. This saturation in cytotoxic response may result from the reduced cellular uptake and self-aggregation behavior of highly hydrophobic compounds. It is well established that optimal lipophilicity is essential for membrane permeability: molecules that are too hydrophilic fail to cross the lipid bilayer, while those that are overly lipophilic may become trapped within the membrane, impairing effective intracellular delivery [80,81]. Moreover, excessive hydrophobicity can lead to self-association and oligomerization of lipopeptides in aqueous environments, reducing the concentration of free monomeric species available for cellular interaction [82,83].

These principles are exemplified by the comparative behavior of compounds **42** and **48**. Compound **42** showed weak cytotoxic activity across all tested cancer cell lines (Figure 1 and Figures S3–S9). In contrast, compound **48**, which incorporates an additional 11-carbon extension compared to **42** (Table S1), exhibited markedly improved cytotoxicity against HeLa cells and, to a lesser extent, HT-29 cells (Table 1). However, no enhancement in activity was observed in B16F10 and PC3 cells. Interestingly, compound **48** also displayed a concentration-dependent decrease in efficacy at higher concentration (80 μM), likely due to self-aggregation induced by its elevated hydrophobicity. These findings underscore the critical importance of maintaining a balanced lipophilic profile in LLP design to maximize cytotoxic potential without compromising bioavailability. Although direct data were not acquired, the dependence of LLP activity on lipophilic tail length and amphiphilic balance suggests a membrane-associated mode of entry, consistent with their observed intracellular effects on caspase activation and oxidative stress.

In addition to lipophilic tail length, the nature of the side chain functional groups played a critical role in modulating the cytotoxic activity of LLPs. Compounds containing tert-butoxycarbonyl (Boc) groups consistently exhibited strong anticancer activity, likely due to their enhanced lipophilicity and favorable membrane interaction properties [84,85]. This is supported by previous reports where Boc-protected amino acid derivatives, particularly sarcosine derivatives, demonstrated both potent anticancer activity and high selectivity toward tumor cells, consistent with our findings that Boc-containing LLPs exhibit potent cytotoxicity with minimal effects on normal cells [86]. In contrast, the substitution of the Boc group with more polar moieties, such as azide or indole, generally led to a reduction in cytotoxic potency. However, notable exceptions were observed with 4-hydroxy-phenyl- and azide-substituted compounds (such as LLPs **53** and **58**) which retained or even enhanced activity in a cell line–dependent manner, suggesting that certain aromatic or electron-rich functionalities can preserve or improve biological function when presented within a compatible structural context.

Phenol-containing compounds have been widely reported for their cytotoxic effects across various cancer cell lines [87,88]. Their mechanism of action may involve the elevation of ROS, particularly hydrogen peroxide, leading to oxidative stress [89,90]. Additional studies have shown that phenolic toxicity can also be expressed during DNA replication, as well as through membrane damage and inhibition of cellular metabolism [91,92,93,94]. Similarly, compounds bearing azide moieties, including LLPs **55**, **58**, and **59**, also demonstrated improved cytotoxic profiles. The cytotoxicity of azide-containing molecules has previously been attributed to their ability to generate ROS and RNS, as well as to their interaction with iron ions in porphyrin complexes, leading to the inhibition of enzymes such as catalase, peroxidases, and cytochrome oxidases [95,96,97]. Importantly, the selectivity of these functionalized LLPs was confirmed through parallel cytotoxicity assays using NIH3T3 embryonic fibroblasts, demonstrating that both phenol- and azide-containing compounds exhibited preferential toxicity toward cancer cells while sparing normal cells.

The mechanistic findings of this study offer valuable insights into how synthetic LLPs exert their anticancer effects. Rather than relying solely on membrane disruption, these compounds act through a multi-faceted mechanism involving the induction of caspase-dependent apoptosis, inhibition of cell proliferation, and generation of reactive oxygen and nitrogen species. Flow cytometry revealed a pronounced accumulation of cells in the sub-G1 phase, accompanied by increased Annexin V/PI staining, indicating the initiation of programmed cell death in B16F10 cells following LLP treatment. These effects were further substantiated by ApoStat-based caspase activation assays and immunocytochemical detection of caspase-3, confirming engagement of the intrinsic (mitochondria-mediated) apoptotic pathway. The lack of autophagic characteristics in LLP-treated cells indicates that these compounds predominantly cause apoptosis, with limited or no activation of autophagy-related cell death mechanisms. This selective activation of apoptosis may reflect the strong pro-apoptotic and oxidative stress–mediated activity of the tested LLPs. Our findings are consistent with previous reports on natural lipopeptides such as surfactin, iturin, fengycin and fellutamides, which have been shown to induce apoptosis via mitochondrial membrane depolarization, elevated ROS production, and the modulation of apoptotic gene expression, specifically, upregulation of pro-apoptotic genes (e.g., BAX, BAD) and downregulation of anti-apoptotic BCL2 [54,58,98,99,100,101,102,103,104], including novel synthetic derivatives and mimics, like peptoids.

Building upon these established mechanisms, our study further demonstrates that synthetic LLPs bearing distinct functional groups—namely Boc, phenol, and azide—differentially modulate intracellular levels of ROS, RNS, and NO in B16F10 cells. This variation in redox response suggests that oxidative imbalance and mitochondrial stress contribute substantially to their cytotoxic mechanisms [105,106]. Notably, elevated production of reactive oxygen and nitrogen species has been observed in many tumor cell types following treatment with lipopeptides, and is known to sensitize cells to apoptosis by damaging DNA, oxidizing membrane lipids, and disrupting redox-sensitive survival pathways [23,101,102,104,107]. The ability of specific LLPs to induce these redox shifts in a structure-dependent manner highlights their potential to be chemically tailored for enhanced pro-apoptotic activity and selective tumor cell targeting. Forthcoming studies will further investigate this relationship by employing ROS scavengers and caspase inhibitors to confirm the mechanistic link between reactive oxygen and nitrogen species production and apoptosis induction in LLP-treated cancer cells.

While this study provides robust mechanistic insights into the anticancer activity of synthetic linear lipopeptides (LLPs), especially of the peptide–peptoid type, several limitations and future opportunities should be considered. All experiments were conducted in vitro, and the absence of in vivo data limits our ability to evaluate the pharmacokinetics, biodistribution, systemic toxicity, and tumor-selective efficacy of these compounds under physiological conditions. Future in vivo studies in suitable animal models will be essential to determine their translational potential. Additionally, although multiple assays confirmed caspase-dependent apoptosis and oxidative imbalance as key mechanisms of action, further validation using genetic or pharmacological modulators, such as caspase inhibitors or ROS scavengers, is warranted to definitively establish pathway involvement. Structure-guided optimization of LLP scaffolds to fine-tune selectivity, potency, and functional group composition may enhance therapeutic indices. Moreover, evaluating combination strategies with conventional chemotherapeutics or targeted agents could open avenues for synergistic cancer treatments. Taken together, these findings provide a strong foundation for the continued development of LLPs as anticancer agents. Future work should emphasize further SAR improvement, in vivo validation and expanded mechanistic exploration across diverse cancer models to fully understand the therapeutic potential.

## 4. Materials and Methods

### 4.1. General

Digitonin was obtained from Riedel-De Haën (Seelze, Germany). MTT, acridine orange, DAPI, crystal violet, surfactin, paraformaldehyde, and Dulbecco’s Modified Eagle’s Medium (DMEM) were purchased from Sigma-Aldrich (Darmstadt, Germany). Roswell Park Memorial Institute (RPMI) 1640, trypan blue, caspase-3 rabbit antibody, and Alexa Fluor 488-conjugated secondary anti-rabbit antibody were obtained from Life Technologies (Darmstadt, Germany). PBS and trypsin-EDTA were purchased from PAN-Biotech (Aidenbach, Germany), and DAF-FM was obtained from Cayman Chemical Company (Hamburg, Germany). Fetal calf serum (FCS) and penicillin/streptomycin (10,000 units per mL penicillin and 100 mg per mL streptomycin) were sourced from PAA Laboratories (Freiburg, Germany). DMSO was purchased from Duchefa Biochemie (Haarlem, The Netherlands), and acetic acid (33%) from Carl Roth GmbH (Karlsruhe, Germany). Apostat was purchased from R & D systems (Minneapolis, MN, USA), DHR, CFSE and Annexin V/PI kits were obtained from BD Biosciences (San Jose, CA, USA).

The investigated LLPs were synthesized via the Ugi four-component reaction as previously reported by Wessjohann and co-workers [31,108]. The identity and purity of the compounds were confirmed using electrospray ionization mass spectrometry (ESI-MS), as well as proton ^1^H and ^13^C nuclear magnetic resonance (NMR) spectroscopy. Stock solutions of all LLPs, digitonin and surfactin were prepared in DMSO at a concentration of 20 mM.

### 4.2. Cell Lines and Culture Conditions

Human prostate cancer cells (PC3) were obtained from the German Collection of Microorganisms and Cell Cultures (DSMZ, Braunschweig, Germany), and human cervical adenocarcinoma cells (HeLa) were obtained from Ontochem (Halle, Germany). Mouse skin melanoma cells (B16F10) and human colon adenocarcinoma cells (HT-29) were kindly provided by Prof. B. Seliger (Department of Immunology, Martin Luther University Halle-Wittenberg, Germany). Mouse embryonic fibroblast cells (NIH3T3) were acquired from the American Type Culture Collection (ATCC, Manassas, VA, USA).

PC3, HeLa, B16F10, and HT-29 cells were cultured in RPMI 1640 medium supplemented with L-glutamine, sodium bicarbonate, 10% FCS, and 1% penicillin–streptomycin. NIH3T3 cells were maintained in DMEM containing 1000 mg/L glucose, L-glutamine, and sodium bicarbonate, also supplemented with 10% FCS and 1% penicillin–streptomycin. All cell lines were grown as adherent monolayers at 37 °C in a humidified incubator with 5% CO_2_. Cells were subcultured upon reaching 85–90% confluence. All experiments involving cell culture were conducted in triplicate. Flow cytometry (FACS) analyses were performed in triplicate, and data were analyzed using BD FACSDiva Software (version 7). Quantitative results are expressed as mean ± SD from three independent experiments. IC_50_ values were determined using SigmaPlot 12.5 software with a four-parameter logistic regression model.

### 4.3. MTT and CV Assays

The MTT and CV assays were employed for initial cytotoxicity screening and determination of half-maximal inhibitory concentrations (IC_50_), as described previously [109]. Cells were seeded in 96-well plates and incubated for 24 h prior to treatment. B16F10, HT-29, HeLa, and NIH3T3 cells were seeded at a density of 2000 cells per well, while PC3 cells were seeded at 6000 cells per well due to their slower proliferation rate.

For fast screening, cancer cells were treated with two concentrations of each LLP (0.1 µM and 10 µM) for 72 h in triplicate, with four replicates in each experiment. For IC_50_ determination, cells were exposed to a serial dilution of the test compounds (ranging from 0.625 to 80 µM) for 72 h. Digitonin was used as a positive control in all assays. Following treatment, absorbance was measured using a microplate reader (SpectraMax, Molecular Devices, San Jose, CA, USA) at 540 nm, with a reference wavelength of 670 nm. Cell viability was calculated as a percentage of untreated control wells.

### 4.4. Cell Cycle Analysis

B16F10 cells were seeded overnight in 6-well plates at a density of 1 × 10^5^ cells per well and treated for 72 h with selected active LLPs either at the IC_50_ or 2 × IC_50_ concentration. Following treatment, both floating and adherent cells were collected, washed with PBS, and fixed in 70% ethanol at 4 °C overnight. Fixed cells were stained with DAPI staining solution (1 µg/mL) for 30 min at 37 °C in the dark, followed by PBS washing to remove excess dye. Flow cytometric analysis was performed using a FACSAria III instrument (BD Biosciences, Eysins, Switzerland). Histograms of cell counts versus fluorescence intensity were generated and cell cycle distribution was determined based on mean fluorescence intensity values.

### 4.5. Apoptosis Evaluation by Annexin V-FITC/PI Staining

B16F10 melanoma cells were seeded in 6-well plates at a density of 1 × 10^5^ cells/well and incubated overnight to allow for cell adherence. Subsequently, cells were treated for 72 h with IC_50_ concentrations of LLPs **17**, **53** and **58**. Following treatment, both floating and adherent cells were harvested and washed with PBS. The cells were then resuspended in 100 μL of Annexin V binding buffer containing 5 μL of Annexin V-FITC and 2 μL of PI per sample. Staining was performed for 15 min at 37 °C in the dark. After staining, cells were washed with PBS and analyzed using a BD FACSAria III flow cytometer (BD Biosciences) to quantify the percentages of apoptotic cells.

### 4.6. ApoStat Caspase Detection Assay

B16F10 cells were seeded in 6-well plates at a density of 1 × 10^5^ cells per well and treated for 72 h with IC_50_ concentrations of selected active LLPs. Post-treatment, cells were harvested and washed with PBS. Cells were then stained with 100 μL of working ApoStat solution, prepared by diluting ApoStat reagent (FITC-VAD-FMK) in PBS containing 5% FCS (99 μL of PBS 5% FCS + 1 μL ApoStat per sample). The staining was performed for 30 min at 37 °C in the dark. Following incubation, cells were washed to remove unbound reagents and analyzed by flow cytometry using a FACSAria III system (BD Biosciences). The fluorescence intensity of FITC was measured to assess caspase production, with increased fluorescence indicating elevated caspase activity.

### 4.7. Immunofluorescence Detection of Cleaved Caspase-3

B16F10 cells (1 × 10^5^ cells/well) were seeded in 6-well plates and treated with selected active LLPs at IC_50_ concentration for 72 h. Following treatment, cells were fixed with 4% paraformaldehyde for 15 min at room temperature, and then permeabilized with 0.25% Triton X-100 in PBS for 10 min. After permeabilization, cells were washed three times with PBS (5 min each), followed by blocking with 10% FCS in PBS containing 0.1% Triton X-100 (PBS-T) for 30 min at room temperature to prevent non-specific antibody binding. Subsequently, cells were incubated overnight at 5–10 °C with rabbit anti-cleaved caspase-3 antibody (1:400 dilution in 1% bovine serum albumin [BSA] in PBS-T). After washing, cells were incubated with Alexa Fluor 488-conjugated anti-rabbit secondary antibody (1:200 dilution in 1% BSA PBS-T) for 30 min at room temperature in the dark. For nuclear staining, cells were incubated with DAPI (1 µg/mL in PBS) for 1 min in the dark, followed by a final PBS wash. Immunofluorescence images were acquired using an EVOS FL Auto Imaging System (Life Technologies).

### 4.8. CFSE-Based Cell Proliferation Assay

B16F10 cells were stained with 1 µM carboxyfluorescein succinimidyl ester (CFSE) for 10 min at 37 °C, followed by treatment with compounds **17**, **53**, or **57** at IC_50_ concentrations for 72 h. After incubation, cells were washed, trypsinized, and analyzed by flow cytometry to assess cell division based on CFSE fluorescence intensity.

### 4.9. Detection of ROS/RNS and NO Production

To assess intracellular ROS/RNS levels, B16F10 cells were first stained with 1 µM DHR for 10 min at 37 °C. Following staining, cells were treated with LLPs **17**, **53** or **58** at their IC_50_ concentrations for 72 h. After treatment, cells were trypsinized, washed with PBS, and analyzed by flow cytometry to quantify DHR fluorescence, indicative of ROS/RNS production. For NO detection, B16F10 cells were treated under identical conditions using the same LLP concentrations. After 72 h, cells were incubated with 5 µM DAF-FM diacetate in RPMI 1640 supplemented with 10% FCS for 1 h at 37 °C. The stain was then deactivated by incubation for 15 min with a serum-free medium. Finally, cells were detached, washed with PBS, and subjected to flow cytometric analysis to assess intracellular NO levels.

### 4.10. Autophagy Analysis by Acridine Orange (AO) Staining

B16F10 cells were seeded and treated with selected active LLPs at IC_50_ concentrations for 72 h. Following treatment, cells were collected and stained with 10 µM AO for 15 min at 37 °C in the dark. After staining, cells were washed with PBS, and the resulting cell pellet was resuspended in 500 µL of PBS. Autophagy-associated acidic vesicular organelles were then analyzed by flow cytometry using a FACSAria III instrument (BD Biosciences).

### 4.11. Statistical Analysis

All statistical analyses were performed in SigmaPlot 12.5 using a two-tailed *t*-test, and statistical significance was defined as *p* < 0.05

## 5. Conclusions

This study systematically evaluated the in vitro cytotoxic potential of 60 synthetic linear lipopeptides (LLPs), mostly peptide–peptoid chimeras, selected from a large library generated via the Ugi four-component reaction, against four cancer cell lines: B16F10, HeLa, HT-29, and PC3. Several synthetic LLPs exhibited potent and selective cytotoxic activity, with significantly lower IC_50_ values than surfactin and minimal toxicity toward normal NIH3T3 fibroblasts. Structure–activity relationship (SAR) analysis identified key structural determinants of anticancer activity, including the presence of a C-terminal ester group, a lipophilic tail of at least 14 carbon atoms, and a core consisting of four amino acid units or two peptide–peptoid moieties. These findings emphasize the importance of achieving a precise structural balance to maximize the therapeutic potential of LLPs. Functional group modifications, such as phenol or azide substitutions, further enhanced cytotoxic potency in a cell line–dependent manner. Compound **17** emerged as a particularly promising candidate, exhibiting strong cytotoxicity and favorable selectivity.

Mechanistically, synthetic LLPs induced caspase-dependent apoptosis, evidenced by sub-G1 accumulation, Annexin V/PI staining, and caspase-3 activation. In addition, selected LLPs triggered intracellular generation of reactive oxygen and nitrogen species, suggesting oxidative imbalance as a contributing factor to their cytotoxic mechanisms.

Taken together, these findings underscore the therapeutic potential of synthetic LLPs as a novel class of anticancer agents. The integration of SAR insights with the versatility of Ugi-based synthesis paves the way for rational design and development of next-generation lipopeptide/lipopeptoid-based therapeutics with improved efficacy and safety. Future work will explore drug formulation strategies to enhance LLP solubility, stability, and delivery, and will also assess their potential in combination with conventional chemotherapeutic or targeted agents to improve therapeutic outcomes.

## Figures and Tables

**Table 1:** IC_50_ value (µM) for cytotoxic LLPs (72 h, CV assay). Data represent mean ± SD from three independent biological experiments, each performed with eight technical replicates per concentration.

Compounds	B16F10	HeLa	HT–29	PC3
**1**	11.5 ± 1.7	16.0 ± 1.1	17.1 ± 4.2	14.8 ± 3.5
**3**	6.1 ± 0.1	6.6 ± 0.3	6.2 ± 0.2	6.1 ± 0.2
**5**	11.7 ± 1.5	14.6 ± 0.8	12.0 ± 1.4	11.1 ± 0.5
**8**	6.8 ± 0.4	16.7 ± 3.9	10.7± 0.9	6.7 ± 0.4
**17**	5.6 ± 0.4	5.6 ± 0.3	4.1 ± 0.8	4.1 ± 0.6
**45**	8.0 ± 0.9	7.9 ± 0.6	7.8 ± 0.9	7.8 ± 0.6
**46**	8.0 ± 0.9	8.0 ± 0.4	8.0 ± 0.4	7.8 ± 0.4
**47**	11.5 ± 1.2	12.3 ± 1.3	9.3 ± 0.5	8.8 ± 0.3
**48**	>80	4.0 ± 0.5	8.6 ± 1.5	>80
**53**	7.7 ± 0.3	19.2 ± 1.8	10.3 ± 0.6	9.6 ± 0.5
**55**	9.9 ± 0.1	15.4 ± 0.6	22.9 ± 0.3	10.5 ± 0.5
**58**	7.4 ± 0.1	7.7 ± 0.2	9.1 ± 0.2	5.8 ± 0.5
**59**	11.1 ± 1.2	9.0 ± 0.1	11.8 ± 1.4	7.5 ± 0.2
Surfactin	40.4 ± 0.3 [35]	76.2 ± 1.6 [36]	46.1 ± 2.6	49.4 ± 1.8

## Data Availability

The original contributions presented in this study are included in the article/Supplementary Materials. Further inquiries can be directed to the corresponding authors.

## Supplementary Materials

The following supporting information can be downloaded at: https://www.mdpi.com/article/10.3390/ph18121840/s1, Figure S1. Chemical structures of LLPs investigated (part I oligomeric peptide–peptoid chimeras). Figure S2. Chemical structures of LLPs investigated (part II oligomeric peptide–peptoid chimeras). Figure S3. Cytotoxicity effects of LLPs against B16F10 cell line (72 h, MTT assay). Figure S4. Cytotoxicity effects of LLPs against HeLa cell line (72 h, MTT assay). Figure S5. Cytotoxicity effects of LLPs against HeLa cell line (72 h, CV assay). Figure S6. Cytotoxicity effects of LLPs against HT-29 cell line (72 h, MTT assay). Figure S7. Cytotoxicity effects of LLPs against HT-29 cell line (72 h, CV assay). Figure S8. Cytotoxicity effects of LLPs against PC3 cell line (72 h, MTT assay). Figure S9. Cytotoxicity effects of LLPs against PC3 cell line (72 h, CV assay). Figure S10. LLPs 17, 53, 58, and Surfactin reduce the survival of B16F10 and HeLa cells in a dose-dependent manner, as determined by MTT and CV assays. Figure S11. Correlation between fatty acid chain length in LLPs 35–44 and cell survival of B16F10 (A) and PC3 (B) cells after 72 h treatment at 10 μM, assessed by CV assay. (C) Structure of the investigated LLPs (*n* = number of CH2-groups). Figure S12. Correlation between cell survival of HeLa (A), HT-29 (B), and PC3 (C) cells after 72 h treatment with 10 μM LLPs (CV assay) and the number of N-substituted dipeptide (peptide–peptoid) repetition units in the LLPs 1, 3, 16, 6, and 33. (D) Structure of the investigated LLPs (m = N-substituted dipeptide (i.e., peptide–peptoid) repetition units). Figure S13. (A) Cell cycle distribution of B16F10 cells treated with 2xIC_50_ concentrations of LLPs 17, 53 or 58 for 72 h, assessed by DAPI staining. (B) Flow cytometric analysis of B16F10 cells treated with IC_50_ concentration of active LLPs for 72 h and stained with AO. Table S1. Chemical structures of LLPs investigated (part 3). Table S2. IC_50_ values (µM) for cytotoxic LLPs (72 h, MTT assay).

## Abbreviations

The following abbreviations are used in this manuscript:

LLP	Linear Lipopeptide/Linear Lipopeptoid
Ugi-4CR	Ugi four-component reaction
SAR	Structure–Activity Relationship
CV	Crystal Violet
LP	Lipopeptide/Lipopeptoid
IC_50_	Half-Maximal Inhibitory Concentration
ApoStat	FITC-conjugated pan-caspase inhibitor
CFSE	Carboxyfluorescein Succinimidyl Ester
AO	Acridine Orange
ROS/RNS	Reactive Oxygen and Nitrogen Species
NO	Nitric Oxide
