# Supplementary material for: Synthetic Linear Lipopeptides and Lipopeptoids Induce Apoptosis and Oxidative Stress: In Vitro Cytotoxicity and SAR Evaluation Against Cancer Cell Lines
Source: Pharmaceuticals (Basel). 2025 Dec 2;18(12):1840. doi: 10.3390/ph18121840 (PMC12735945; doi:10.3390/ph18121840)
Supplement: Supplementary file 1 [file pharmaceuticals-18-01840-s001.zip › pharmaceuticals-3977049-supplementary.pdf]

# Synthetic Linear Lipopeptides and Lipopeptoids Induce Apoptosis and Oxidative Stress: In Vitro Cytotoxicity and SAR Evaluation against Cancer Cell Lines

**Ali Hmedat [1,2], Sebastian Stark [3], Tuvshinjargal Budragchaa [3], Nebojša Đ. Pantelić [4], Ludger A. Wessjohann [3,*] and Goran N. Kaluđerović [1,3,*]**

[1] Department of Engineering and Natural Sciences, University of Applied Sciences Merseburg, Eberhard-Leibnitz-Strasse 2, 06217 Merseburg, Germany; goran.kaluderovic@hs-merseburg.de (GNK)
[2] Department of Pharmaceutics and Pharmaceutical Technology, Faculty of Pharmacy, Yarmouk University, Irbid 21163, Jordan; ali.hmedat@yu.edu.jo (AH)
[3] Department of Bioorganic Chemistry, Leibniz Institute of Plant Biochemistry,Weinberg 3, 06120 Halle (Saale), Germany; starksebastian82@gmail.com (SS), Tuvshinjargal.Budragchaa@ipb-halle.de (TB), Ludger.Wessjohann@ipb-halle.de (LAW)
[4] Department of Chemistry and Biochemistry, Faculty of Agriculture, University of Belgrade, Nemanjina 6, 11080 Belgrade, Serbia; pantelic@agrif.bg.ac.rs (NP)
* Correspondence: goran.kaluderovic@hs-merseburg.de

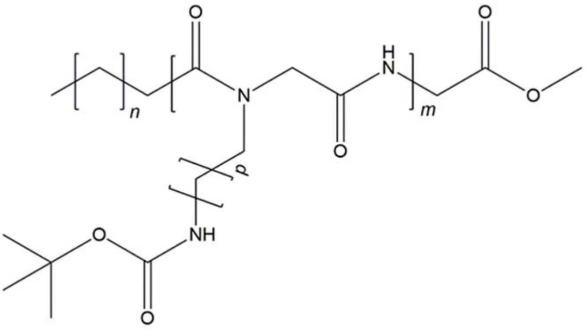


| Comopounds | *n* | *m* | *p* | Compounds | *N* | *m* | *p* | Compounds | *n* | *m* | *p* |
|---|---|---|---|---|---|---|---|---|---|---|---|
| **18** | 7 | 1 | 3 | **36** | 7 | 2 | 2 | **47** | 11 | 2 | 3 |
| **19** | 8 | 1 | 3 | **37** | 8 | 2 | 2 | **5** | 13 | 2 | 3 |
| **20** | 9 | 1 | 3 | **38** | 9 | 2 | 2 | **45** | 14 | 2 | 3 |
| **11** | 12 | 1 | 3 | **39** | 10 | 2 | 2 | **46** | 15 | 2 | 3 |
| **9** | 13 | 1 | 3 | **40** | 11 | 2 | 2 | **24** | 1 | 2 | 5 |
| **12** | 14 | 1 | 3 | **41** | 12 | 2 | 2 | **17** | 11 | 2 | 5 |
| **13** | 15 | 1 | 3 | **42** | 13 | 2 | 2 | **3** | 13 | 2 | 5 |
| **23** | 10 | 1 | 5 | **43** | 14 | 2 | 2 | **8** | 11 | 3 | 5 |
| **10** | 11 | 1 | 5 | **44** | 15 | 2 | 2 | **16** | 13 | 3 | 5 |
| **1** | 13 | 1 | 5 | **28** | 7 | 2 | 3 | **6** | 13 | 4 | 5 |
| **34** | 1 | 2 | 2 | **29** | 8 | 2 | 3 | **33** | 13 | 5 | 5 |
| **35** | 6 | 2 | 2 | **30** | 9 | 2 | 3 | | | | |

**Figure S1. Chemical structures of LLPs investigated (part I oligomeric peptide-peptoid chimeras).**

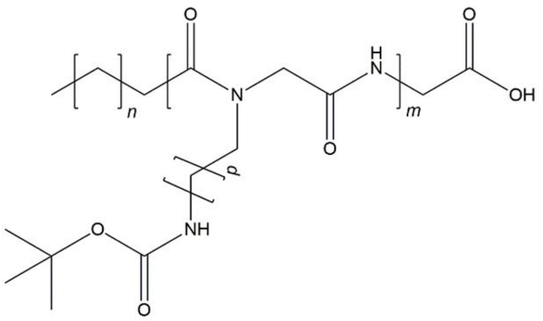

| Compounds | *n* | *m* | *p* |
|---|---|---|---|
| **25** | 7 | 1 | 3 |
| **26** | 8 | 1 | 3 |
| **27** | 9 | 1 | 3 |
| **15** | 1 | 1 | 5 |
| **22** | 11 | 2 | 5 |
| **14** | 13 | 2 | 5 |
| **21** | 13 | 3 | 5 |

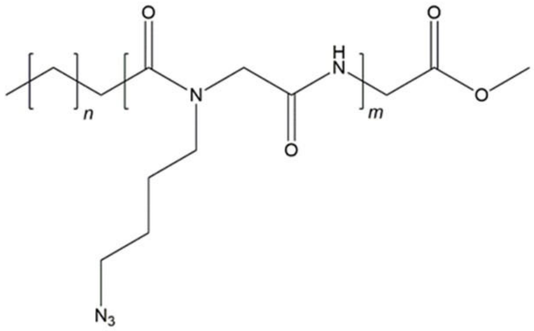

| Compounds | *n* | *m* |
|---|---|---|
| **52** | 13 | 1 |
| **57** | 17 | 1 |
| **55** | 13 | 2 |
| **58** | 16 | 2 |
| **59** | 17 | 2 |
| **60** | 13 | 3 |

**Figure S2. Chemical structures of LLPs investigated (part II oligomeric peptide-peptoid chimeras).**

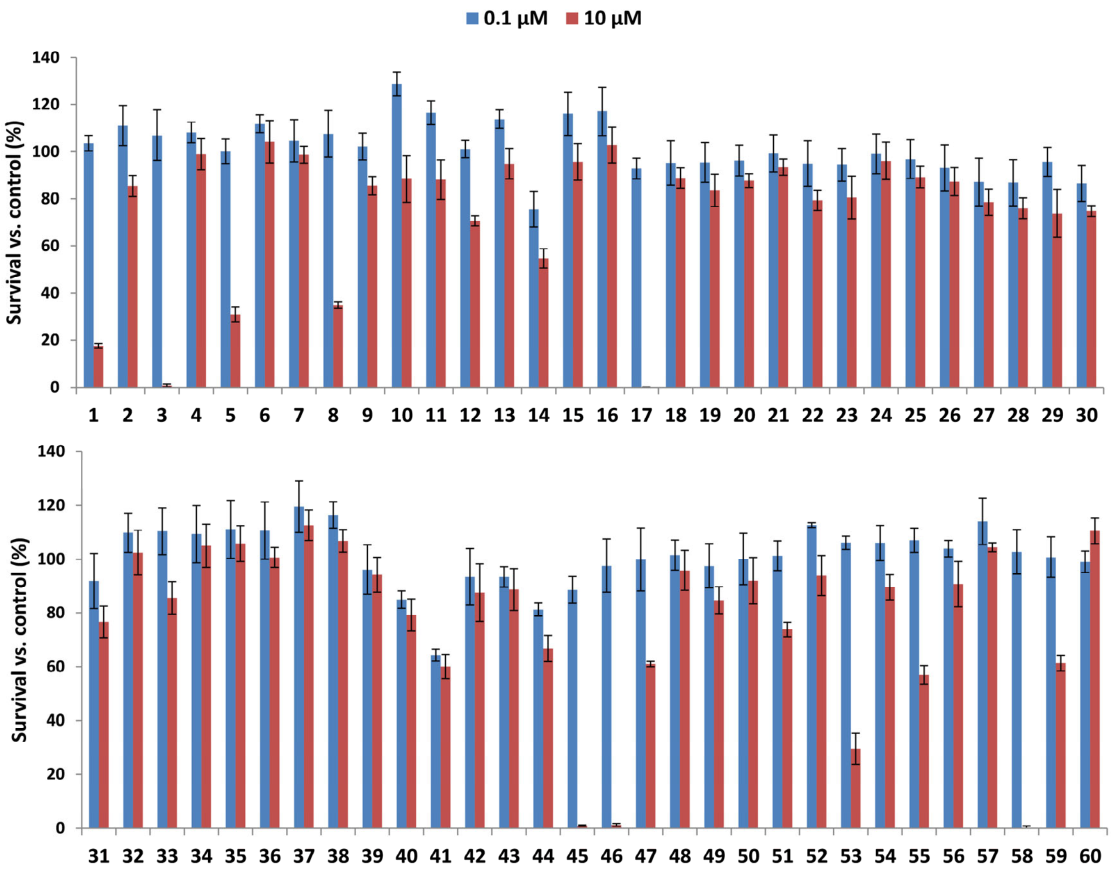


**Figure S3. Cytotoxicity effects of LLPs against B16F10 cell line (72 h, MTT assay).** Cell viability was normalized to DMSO-treated control cells, and data are presented as mean ± SD from three independent biological experiments.

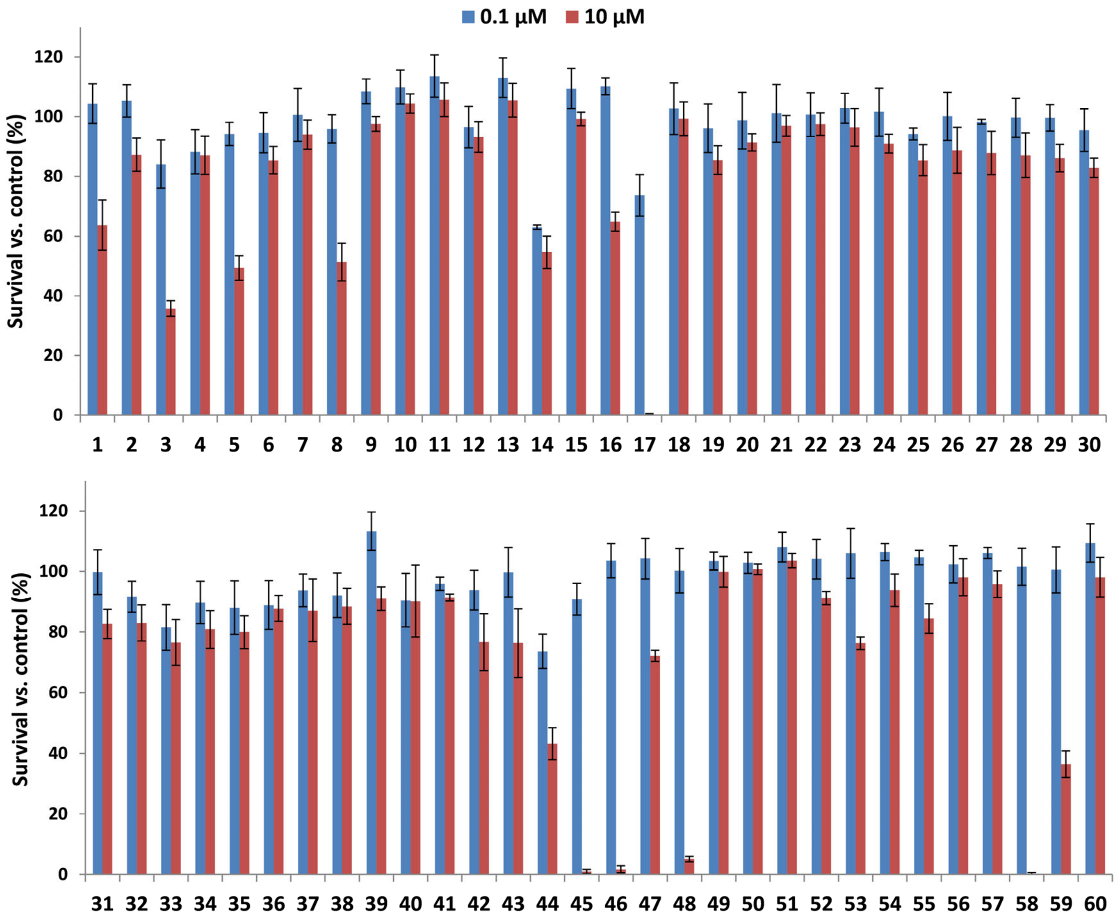


**Figure S4. Cytotoxicity effects of LLPs against HeLa cell line (72 h, MTT assay).** Cell viability was normalized to DMSO-treated control cells, and data are presented as mean ± SD from three independent biological experiments.

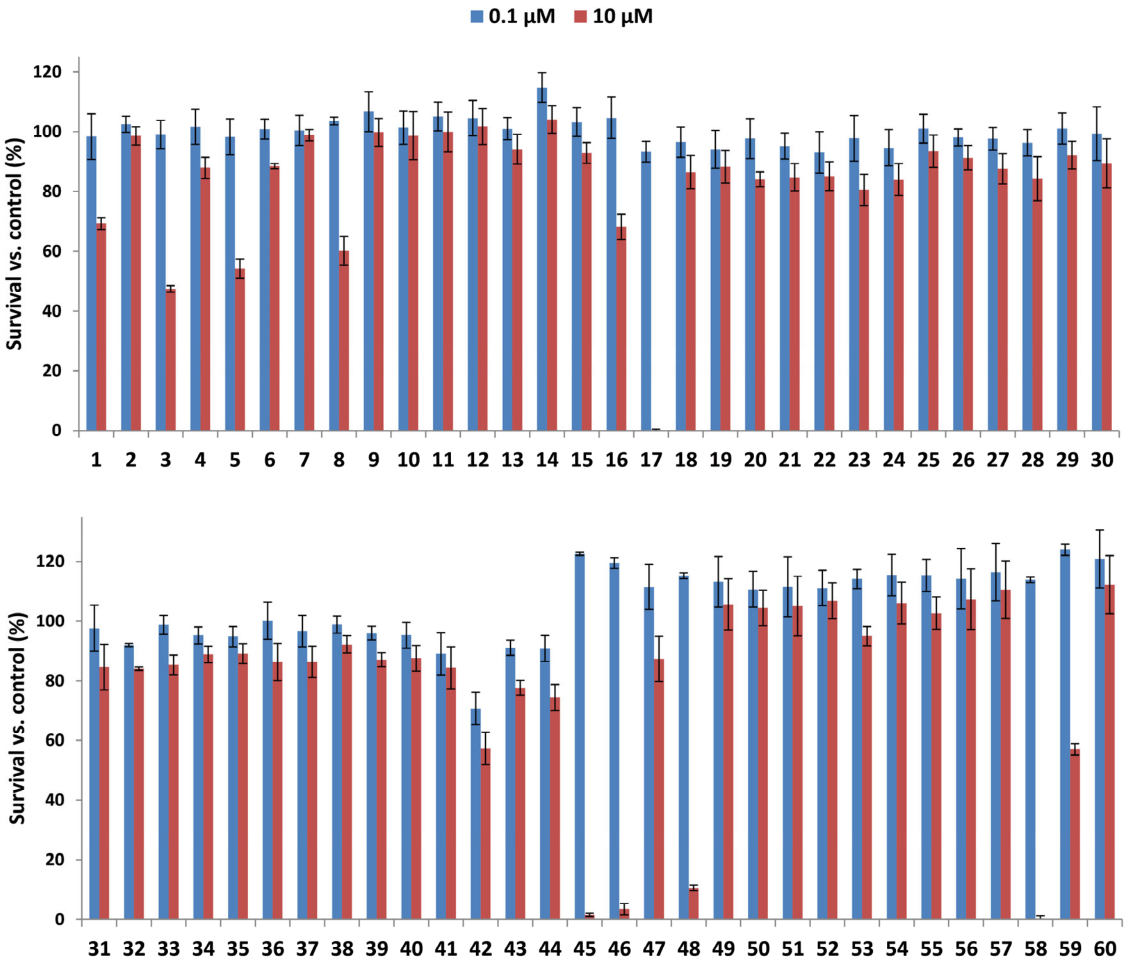


**Figure S5. Cytotoxicity effects of LLPs against HeLa cell line (72 h, CV assay).** Cell viability was normalized to DMSO-treated control cells, and data are presented as mean ± SD from three independent biological experiments.

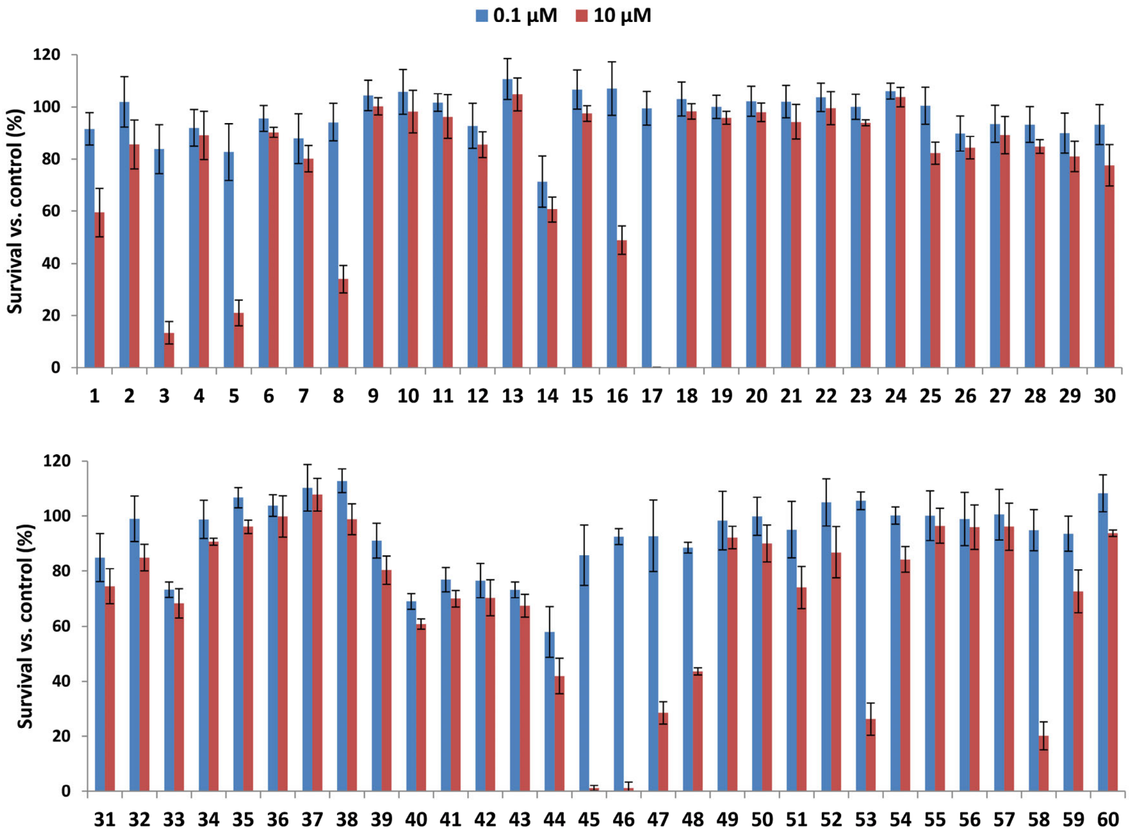


**Figure S6. Cytotoxicity effects of LLPs against HT-29 cell line (72 h, MTT assay).** Cell viability was normalized to DMSO-treated control cells, and data are presented as mean ± SD from three independent biological experiments.

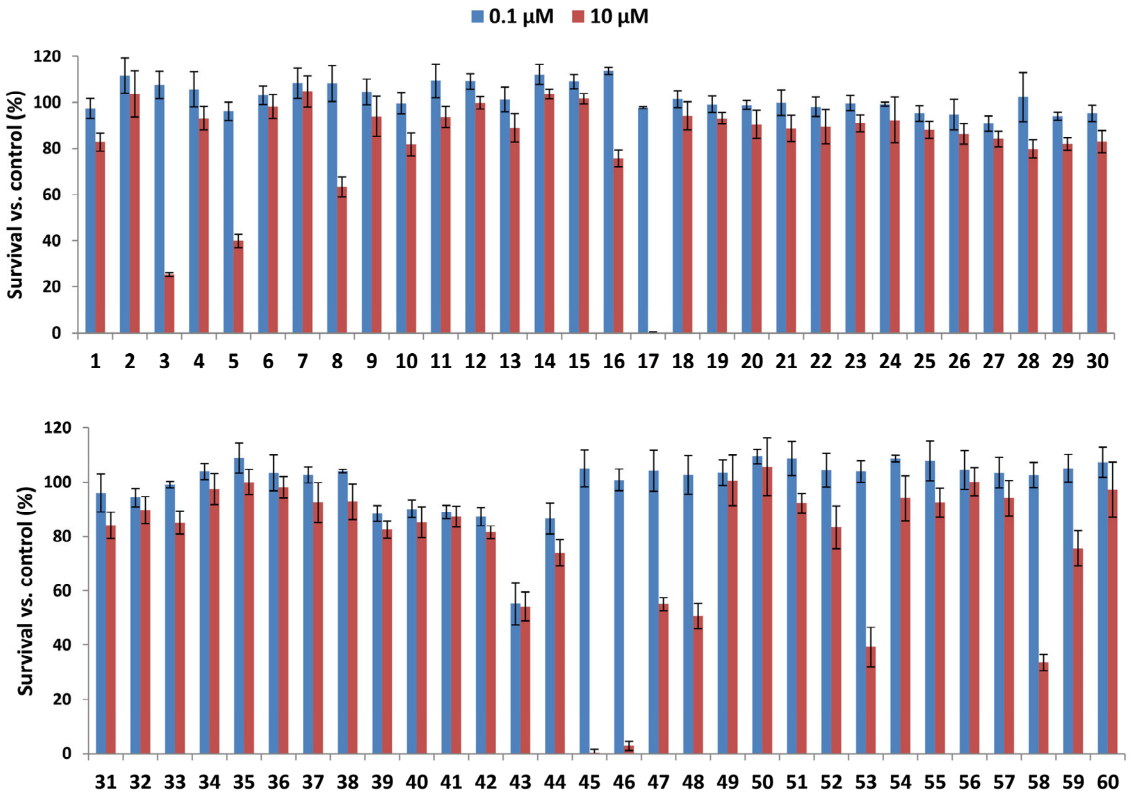


**Figure S7. Cytotoxicity effects of LLPs against HT-29 cell line (72 h, CV assay).** Cell viability was normalized to DMSO-treated control cells, and data are presented as mean ± SD from three independent biological experiments.

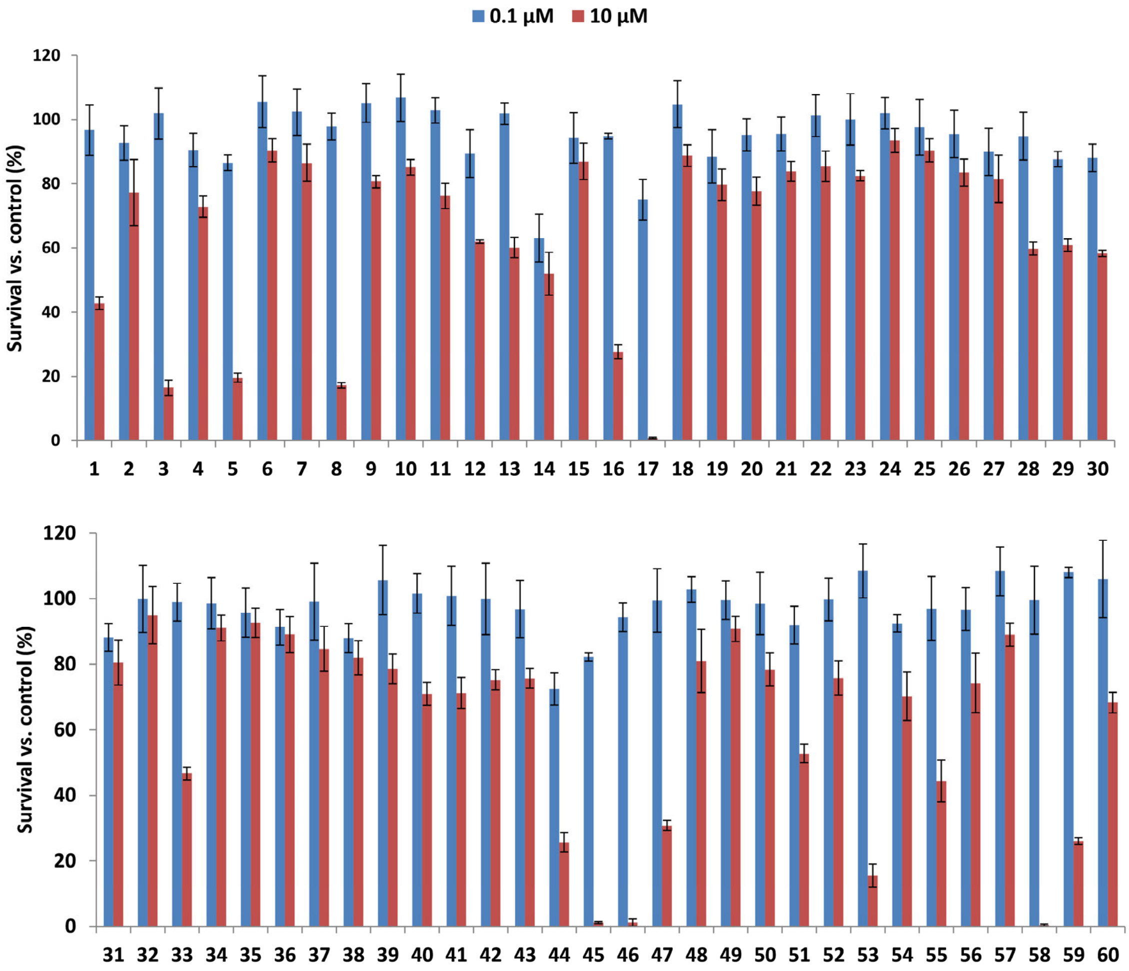


**Figure S8. Cytotoxicity effects of LLPs against PC3 cell line (72 h, MTT assay).** Cell viability was normalized to DMSO-treated control cells, and data are presented as mean ± SD from three independent biological experiments.

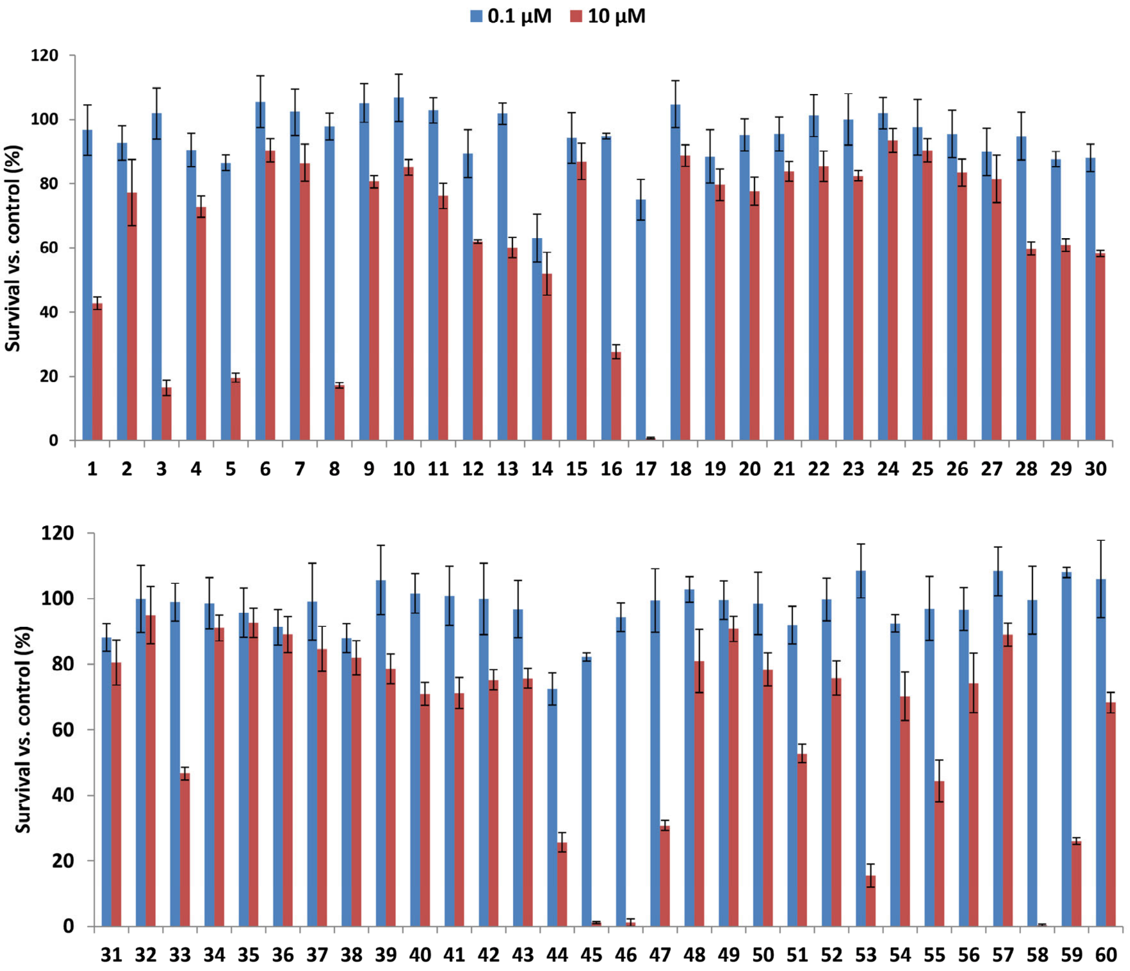


**Figure S9. Cytotoxicity effects of LLPs against PC3 cell line (72 h, CV assay).** Cell viability was normalized to DMSO-treated control cells, and data are presented as mean ± SD from three independent biological experiments.

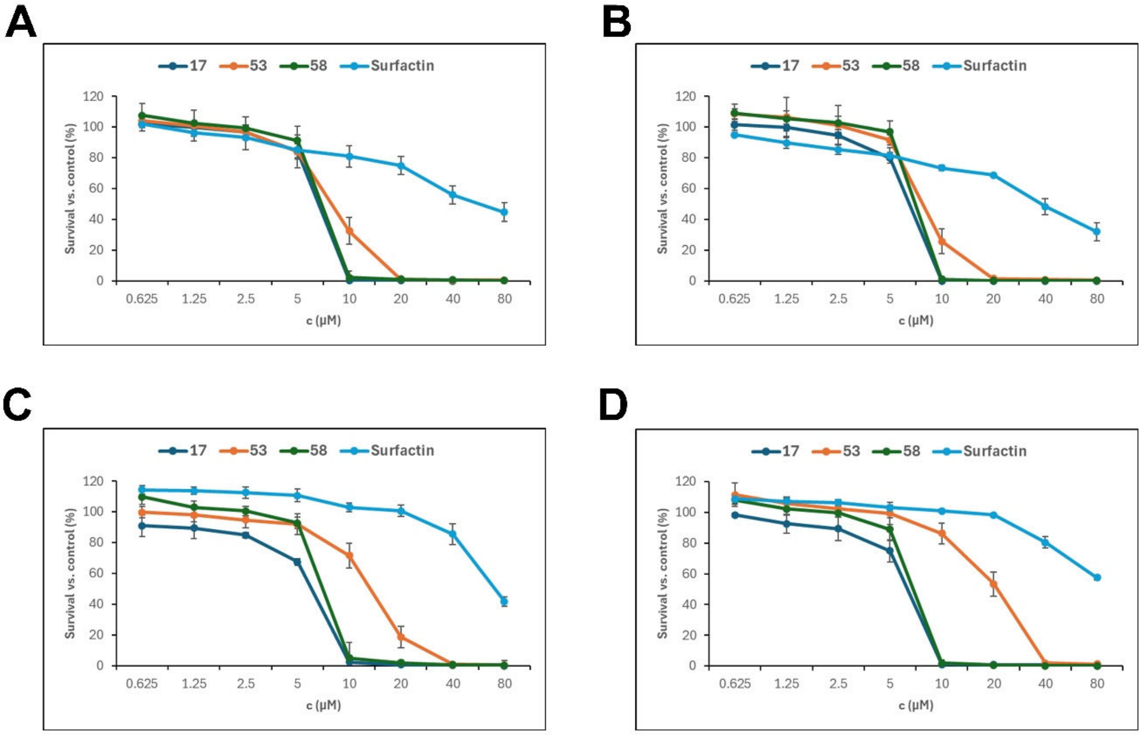


**Figure S10**. **LLPs 17, 53, 58, and surfactin reduce the survival of B16F10 and HeLa cells in a dose-dependent manner, as determined by MTT and CV assays.** Cells were treated with varying concentrations (0.0625–80 µM) of each compound for 72 h. Cell viability was normalized to DMSO-treated controls, and dose-response curves are presented as mean ± SD from three independent biological replicates. (A) B16F10, MTT assay; (B) B16F10, CV assay; (C) HeLa, MTT assay; and (D) HeLa, CV assay.

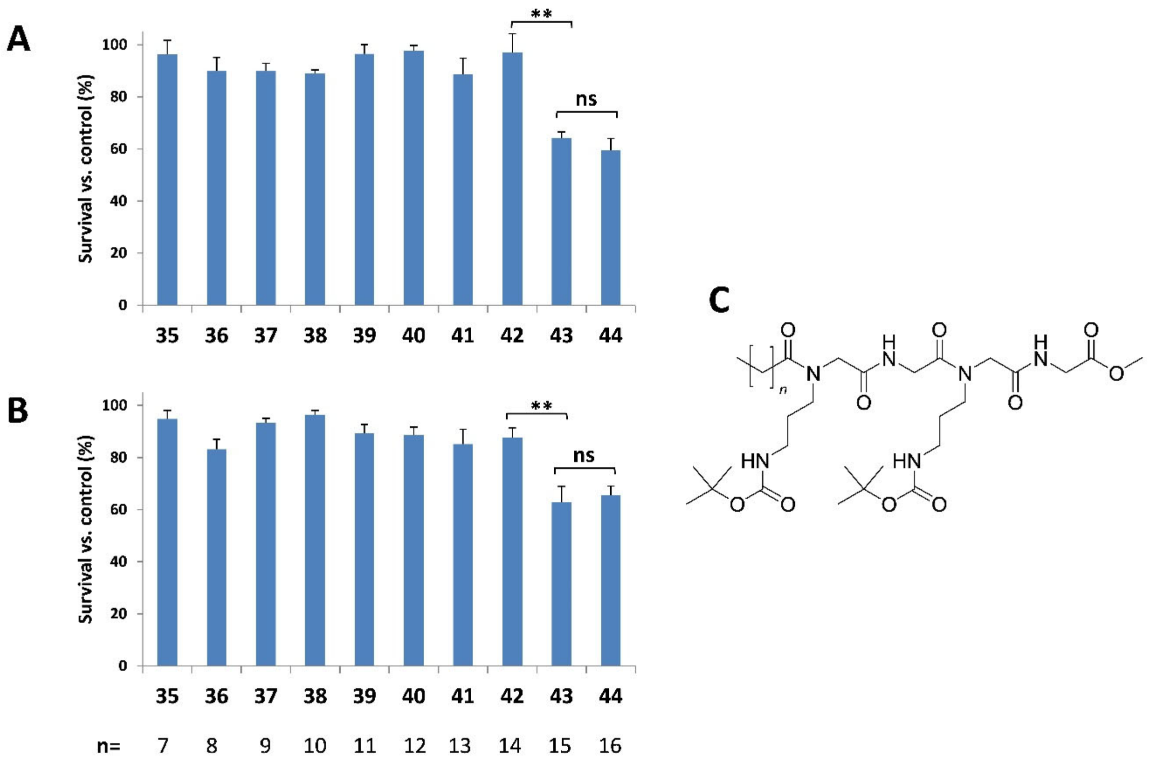


**Figure S11. Correlation between fatty acid chain length in LLPs 35–44 and cell survival of B16F10 (A) and PC3 (B) cells after 72 h treatment at 10 µM, assessed by CV assay.** Values are normalized to DMSO-treated cells and are presented as mean ± SD from three independent experiments. Statistical significance was determined using a two-tailed *t*-test (ns = not significant; ** $p < 0.01$). **(C) Structure of the investigated LLPs (n = number of $CH_2$ groups).**

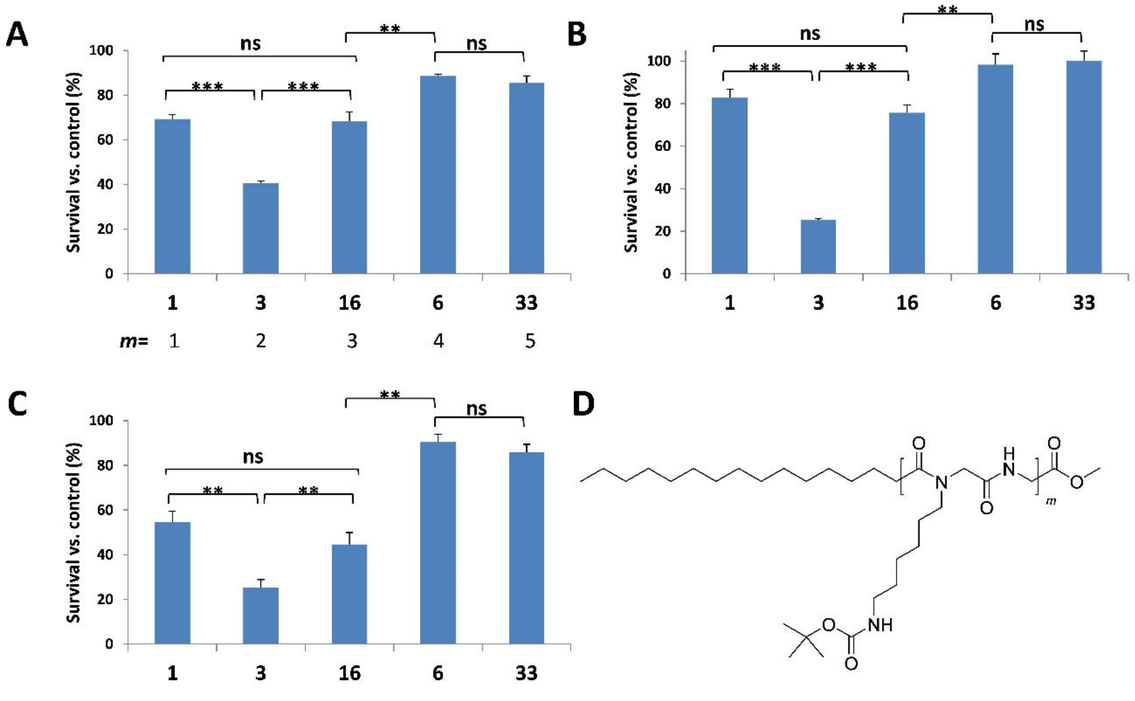


**Figure S12. Correlation between cell survival of HeLa (A), HT-29 (B), and PC3 (C) cells after 72 h treatment with 10 µM LLPs (CV assay) and the number of *N*-substituted dipeptide (peptide-peptoid) repetition units in the LLPs 1, 3, 16, 6, and 33.** Values are normalized to DMSO-treated cells and are presented as mean ± SD from three independent experiments. Statistical significance was assessed using a two-tailed *t*-test. Significance levels: ns = not significant; ** $p < 0.01$; *** $p < 0.001$. **(D) Structure of the investigated LLPs (m = *N*-substituted dipeptide (i.e. peptide-peptoid) repetition units).**

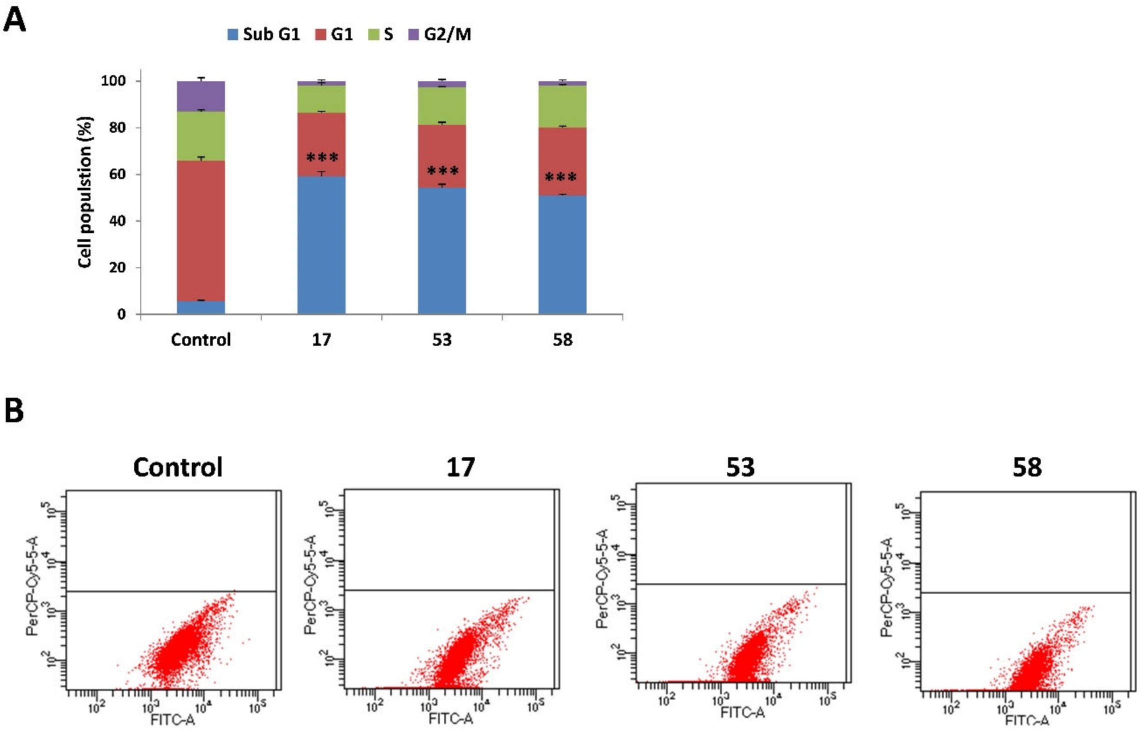


**Figure S13. (A) Cell cycle distribution of B16F10 cells treated with 2x$IC_{50}$ concentrations of LLPs 17, 53 or 58 for 72 h, assessed by DAPI staining.** Values are shown as mean ± SD from three independent experiments, and statistical significance versus control was determined using a two-tailed *t*-test ($p < 0.001$, **). **(B) Flow cytometric analysis of B16F10 cells treated with the $IC_{50}$ concentration of active LLPs for 72 h and stained with AO.**

**Table S1. Chemical structures of LLPs investigated (part 3).**

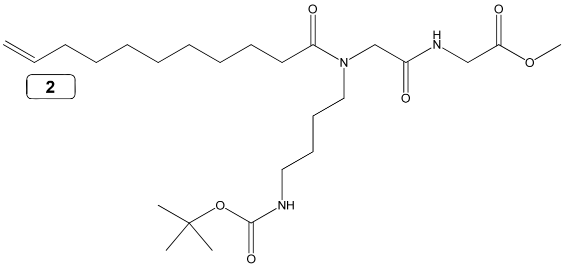

2

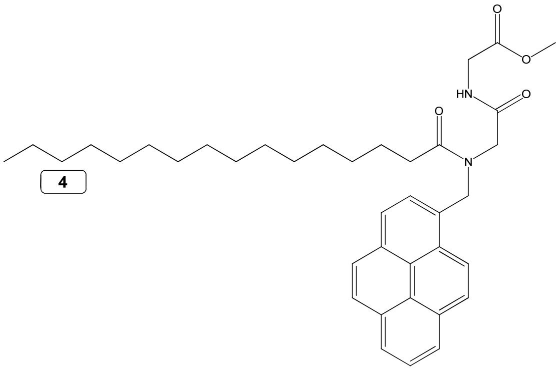

4

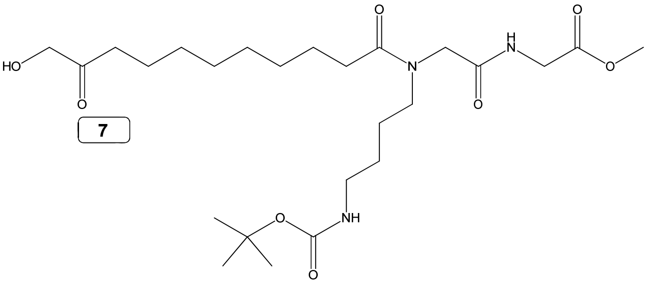

7

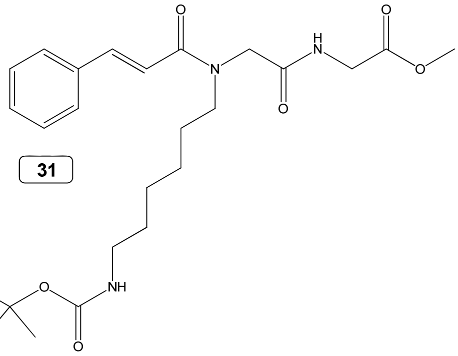

31

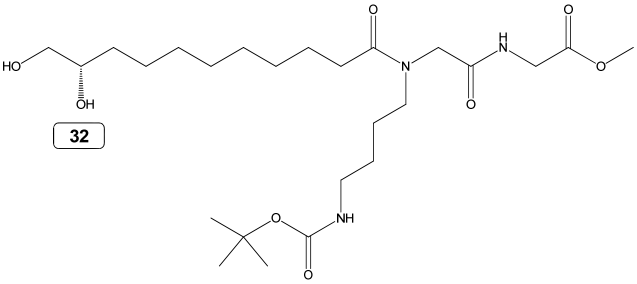

32

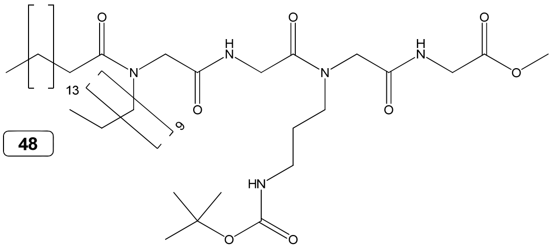

48

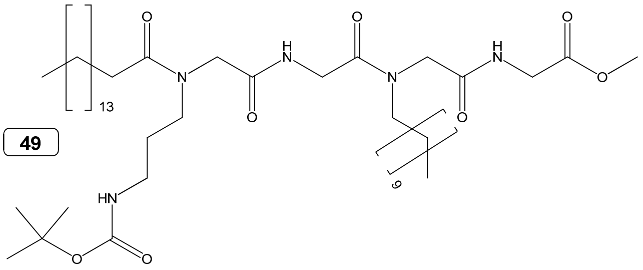

49

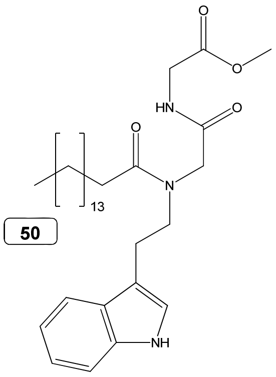

50

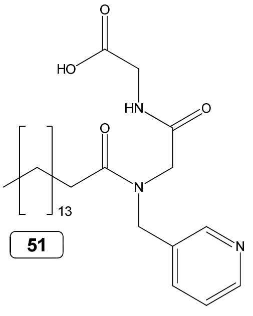

51

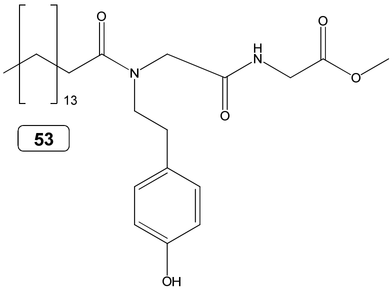

53

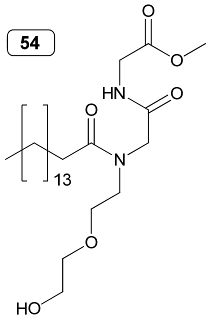

54

**Table S2. IC$_{50}$ values (µM) for cytotoxic LLPs (72 h, MTT assay).** Data represent mean ± SD from three independent biological experiments.

| Compounds | B16F10 | HeLa | HT-29 | PC3 |
|---|---|---|---|---|
| **1** | 9.9 ± 1.2 | 22.1 ± 1.9 | 23.3 ± 4.2 | 16.0 ±0.4 |
| **3** | 6.1 ± 0.1 | 6.4 ± 0.9 | 6.5 ± 0.6 | 5.9 ± 0.1 |
| **5** | 12.4 ± 0.9 | 12.5 ± 0.5 | 11.2 ± 0.7 | 9.3 ± 0.6 |
| **8** | 5.2 ± 0.6 | 15.1 ± 4.6 | 6.1 ± 0.4 | 4.1 ± 0.3 |
| **17** | 5.7 ± 0.3 | 5.6 ± 0.3 | 4.0 ± 0.6 | 4.0 ± 0.3 |
| **45** | 8.0 ± 0.2 | 8.0 ± 0.5 | 7.5 ± 0.1 | 6.7 ± 0.6 |
| **46** | 8.1 ± 0.1 | 8.2 ± 0.3 | 7.4 ± 0.3 | 7.0 ± 0.5 |
| **47** | 12.1 ± 2.1 | 12.3 ± 0.8 | 7.8 ± 0.4 | 7.6 ± 0.4 |
| **48** | > 80 | 3.6 ± 0.2 | 5.5 ± 1.1 | 14.7 ± 0.5 |
| **53** | 8.0 ± 0.2 | 13.2 ± 0.4 | 9.7 ± 0.2 | 7.8 ± 0.3 |
| **55** | 10.9 ± 0.3 | 15.4 ± 0.4 | 21.2 ± 0.7 | 9.2 ± 0.1 |
| **58** | 7.5 ± 0.2 | 7.8 ± 0.3 | 8.7 ± 0.3 | 5.4 ± 0.4 |
| **59** | 11.5 ± 1.3 | 11.5 ± 1.0 | 12.4 ± 0.3 | 6.1 ± 0.3 |
| **surfactin** | 50.3 ± 0.6 | 64.9 ± 0.8 | 54.4 ± 1.9 | 57.7 ± 2.7 |
